# Development of a digital application to train and debrief situational awareness in interprofessional teams: A simulation-based approach to mitigate patient safety hazards with a virtual room of error

**DOI:** 10.3205/zma001818

**Published:** 2026-02-17

**Authors:** Veronika Spielmann, Margrit Ebinger, Christina Jaki

**Affiliations:** 1 Stuttgart, Germany; 2 DHBW Stuttgart, Stuttgart, Germany; 3 Klinikum Stuttgart, Simulationszentrum STUPS, Stuttgart, Germany

**Keywords:** patient safety, awareness, medical errors, simulation training, software

## Abstract

**Objective:**

This project between DHBW Stuttgart and the simulation center at Klinikum Stuttgart (STUPS) addressed the following question: How can interprofessional teams be virtually trained and debriefed for situational awareness (SA) to mitigate patient safety hazards? The objective was to develop a digital, simulation-based application for both training and debriefing SA in interprofessional teams.

**Method:**

Using Design Science Research (DSR), a software application was iteratively developed based on literature and stakeholder-informed objectives. These guided the development of a prototypical version, which was demonstrated and tested with a small group of interprofessional teams to assess real-world applicability. Participant feedback informed subsequent refinement into the fully developed application.

**Results:**

The resulting web-based application combines simulation-based training and structured debriefing on a single platform for trainers and participants. It offers a virtual implementation of the Room of Error (ROE), enabling team-based SA training in near-real scenarios with embedded patient safety hazards, gamification, and real-time interprofessional collaboration (IPC). Structured debriefing is embedded in the workflow and supported by automated tracking and evaluation tools to help trainers assess performance and lead reflective discussions.

**Conclusion:**

The developed application offers a practical solution for training and debriefing SA in interprofessional teams via a virtual ROE. It contributes to education efforts by combining experiential learning with structured reflection, aligning with global patient safety and medical education priorities. While feasibility has been demonstrated, further research is needed to empirically assess its impact on SA and IPC.

## Zusammenfassung

**Zielsetzung:**

Dieses Projekt zwischen der DHBW Stuttgart und dem Simulationszentrum am Klinikum Stuttgart (STUPS) befasste sich mit der folgenden Frage: Wie kann Situationsbewusstsein (SB) in interprofessionellen Teams virtuell trainiert und nachbesprochen werden, um Risiken für die Patientensicherheit zu reduzieren? Ziel war es, eine digitale, simulationsbasierte Anwendung für das Training und Debriefing von SB in interprofessionellen Teams zu entwickeln.

**Methode:**

Unter Anwendung des Design Science Research (DSR)-Ansatzes wurde eine Softwareanwendung iterativ auf Grundlage von Literatur und unter Einbezug der Zielsetzungen relevanter Stakeholder entwickelt. Diese leiteten die Entwicklung einer prototypischen Version, welche mit einer kleinen Gruppe von interprofessionellen Teams demonstriert und getestet wurde, um die Anwendbarkeit in der Praxis zu bewerten. Das Feedback der Teilnehmer:innen floss anschließend in die Weiterentwicklung zur finalen Anwendung ein.

**Ergebnisse:**

Die entstandene webbasierte Anwendung vereint simulationsbasiertes Training und strukturiertes Debriefing auf einer gemeinsamen Plattform für Trainer:innen und Teilnehmer:innen. Sie bietet eine virtuelle Umsetzung des Room of Error (ROE) und ermöglicht teambasiertes SB-Training in realitätsnahen Szenarien mit eingebetteten Gefahren für die Patientensicherheit, Gamification und interprofessioneller Zusammenarbeit (IPZ) in Echtzeit. Das strukturierte Debriefing ist in den Ablauf eingebettet und wird durch automatische Tracking- und Auswertungstools unterstützt, die Trainer:innen bei der Leistungsbewertung und der Durchführung von Reflexionsgesprächen unterstützen.

**Fazit:**

Die entwickelte Anwendung bietet eine praxisnahe Lösung zum Training und Debriefing von SB in interprofessionellen Teams mittels eines virtuellen ROE. Sie leistet einen Beitrag zur Aus- und Weiterbildung, indem sie erfahrungsbasiertes Lernen mit strukturierter Reflexion verbindet und sich an den globalen Prioritäten der Patientensicherheit und der medizinischen Bildung orientiert. Während die Umsetzbarkeit bereits demonstriert werden konnte, sind weitere Forschungen erforderlich, um den Einfluss auf SB und IPZ empirisch zu bewerten.

## 1. Introduction

The global imperative to enhance patient safety, driven by significant mortality and financial costs of unsafe care [1], [2], [3], is exemplified in the WHO's Global Patient Safety Action Plan 2021-2030, which envisions “[a] world in which no one is harmed in health care […]” ([4] p.8). Many medical errors stem from human factors, particularly in dynamic healthcare settings [5], [6], [7], [8], [9], [10]. The shift towards digital methodologies, accelerated by COVID-19, offers new opportunities and challenges for patient safety training. Especially human-centred digital technologies can enhance patient safety by improving information and communication, leading the WHO to recommend applying human factors approach to hardware and software applications [2], [4]. 

Given that many errors are rooted in human factors, addressing and mitigating poor practices and medical mistakes is essential for improving patient safety [11], [12], [13], [14], [15], [16], [17], [5]. Often, these errors stem from inadequate organizational culture and deficiencies in non-technical skills (NTS), rather than a lack of knowledge or technical ability [12], [16], [18], [19], [20]. A key NTS is situational awareness (SA), which “[…] facilitates clinical reasoning, diagnostic accuracy, and appropriate goal-directed performance, and enables clinicians to immediately adapt treatment strategies in response to changes in clinical situational actualities” ([21] p.1). SA is vital for safe medical practice, and its absence often leads to errors [11], [22], [23], [24], [25], [26]. Especially in dynamic, complex environments, functioning under time pressure is essential to enhance clinical performance and reduce errors [22], [23], [27], [28]. Hence, SA has been emphasized in both conceptual and empirical studies for its role in preventing adverse events, often tied to perceptual errors [16], [17], [29], [30], [6].

SA is also integral to interprofessional collaboration (IPC), defined to occur “[…] when multiple health workers from different professional backgrounds provide comprehensive services […] to deliver the highest quality of care across settings” ([31] p.7). For example, research underscores that a team’s SA is only as strong as its weakest member [11], and studies confirm that IPC and experiential learning improve team dynamics, decision-making, and error reduction [8], [9]. While much research has focused on improving individual SA [11], [23], [7], growing evidence supports the integration of team-based training, which significantly impacts patient safety [7], [32], [10]. Notably, interprofessional learning has been shown to reduce patient mortality [33]. A shift towards team-focused interventions is therefore essential.

A validated approach to translate this into practice is training in human factors, which strengthens interpersonal skills, communication, teamwork, and clinical competencies [23], [32], [34], [35], [36]. Research supports team-based interventions to enhance SA and patient safety, emphasizing experiential learning, continuous reflection, and mutual respect [7], [8], [9], [37], [38]. Training-based approaches also align with the WHO’s objective of integrating patient safety into professional education, emphasizing interprofessional team training [4], [31], [39]. Recent technological advancements have accelerated the adoption of simulation-based education, which allows skill development in a controlled environment and promotes professional growth without risking patient safety [40], [41], [42], [43], [44], [45], [46]. Given its potential to enhance patient safety across healthcare professions, it is predicted to become standard [45], [47]. Studies highlight the benefits of simulation in raising clinical awareness, fostering safe care, and enhancing learning through debriefing [48], [49]. As a central element of simulation-based training, debriefing is critical for improving teamwork, fostering a learning culture, and advancing healthcare quality [50], [51], [52], [40]. By promoting critical reflection and analysis, it is considered fundamental to effective simulation-based education [41], [53]. 

This underscores the synergistic potential of interprofessional, simulation-based training combined with active reflection. Despite its recognized value, the WHO emphasizes that “[…] education and training of health care professionals has been underused and undervalued as a vital tool to address the challenges of achieving improved patient safety […]” ([4] p.49). Prior studies show that the Room of Error (ROE), also known as the Room of Horror, is increasingly applied in healthcare training [54], [55], [56], [57], [58], [47], [59]. Zimmermann et al. [56] evaluated it as a low-fidelity SA simulation in 13 Swiss hospitals, where participants detected fewer than half of the embedded hazards – highlighting limitations in existing SA training. Interprofessional group interactions, however, enhanced error detection, stressing the value of collaborative components. The study confirms the importance of structured debriefing and team-based formats. Addressing physical format limits, Mascarenhas et al. [60] developed a 3D virtual ROE, which improved accessibility and satisfaction. Yet, their externally developed, individual-focused tool lacked adaptability, team collaboration, and integrated debriefing. These gaps are echoed in a systematic review by Jung et al. [58].

These limitations reveal a broader gap: the lack of digital solutions that integrate experiential, team-based learning under realistic conditions with structured debriefing. To address this, the report presents the development of a digital, simulation-based application guided by the research question: How can interprofessional teams be virtually trained and debriefed for SA to mitigate the risk of patient safety hazards? The objective was to develop a simulation-based application for both training and debriefing SA in interprofessional teams. 

Using Design Science Research (DSR) as our methodological framework, the software development is detailed. The results show how the application provides a holistic solution by integrating simulation-based training with structured debriefing to promote IPC and SA in realistic scenarios. The report ends with a critical discussion of the findings and a conclusion. 

## 2. Methodology

To address the research question, we employed DSR: a methodology from information systems research focused on developing and evaluating technology-based artifacts to solve real-world challenges [61], [62], [63]. Our artifact is a simulation-based application enabling interprofessional teams to be virtually trained and debriefed in SA to help mitigate patient safety hazards in a ROE setting. DSR aligns with WHO principles for designing safe, resilient patient safety systems by integrating human factors, multidisciplinary collaboration, and the sociotechnical environment [4]. 

**Figure 1:**
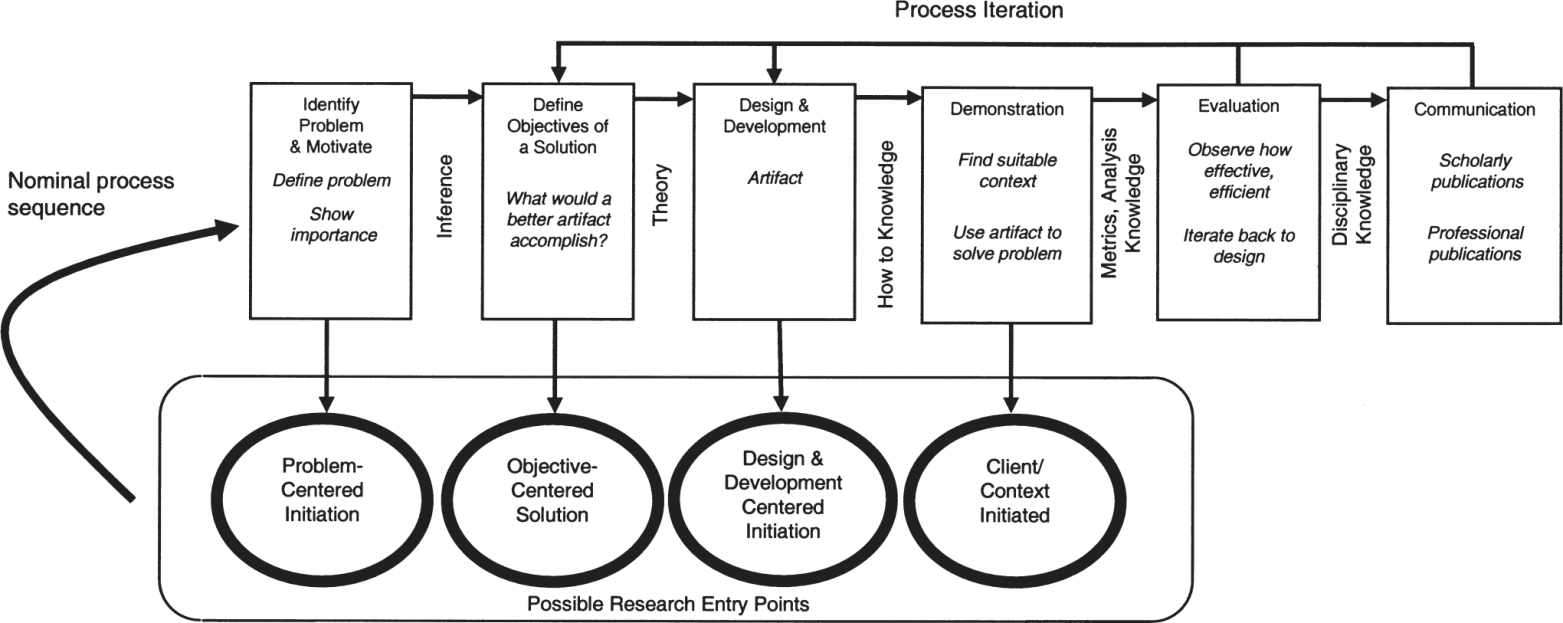
DSR methodology according to Peffers et al. [63], p.54

We followed the six-step model proposed by Peffers et al. ([63] p.54) (see figure 1 (Fig. 1)), which provides a structured and iterative approach to DSR:

1. Problem identification and motivation: The project is motivated by the high incidence of preventable medical errors and the lack of a comprehensive solution for training and debriefing SA in interprofessional teams.
2. Definition of objectives for a solution: Based on literature and stakeholder input, we defined objectives for a solution supporting both SA training and debriefing in interprofessional teams (see chapter 3).
3. Design and development: We developed a digital application that simulates realistic clinical scenarios with a virtual ROE and facilitates team-based SA training and integrated debriefing. A first prototypical version was iteratively refined based on user feedback, resulting in the second version presented in this report (see chapter 4).
4. Demonstration: The prototypical application was tested in a session with interprofessional teams composed of final-year medical, health and nursing sciences students.
5. Evaluation: Feedback from the demonstration was collected and analysed. Insights informed the refinement into the second version of the application.
6. Communication: This report presents the DSR results for dissemination among educators, researchers, and other stakeholders to support adoption and further research.

## 3. Development of a digital, simulation-based application

The application was developed in a collaborative project between the Cooperative State University (DHBW) Stuttgart [64] and the simulation center at the Klinikum Stuttgart (STUPS) [65]. STUPS offers simulation training focused on patient safety and operates a physical ROE (see attachment 1 , appendix 1), where participants identify patient safety hazards in a simulated clinical setting [54], [55], [56], [57]. Early efforts to digitize the format - using a digital picture board in classroom settings [https://padlet.com/] (see attachment 1 , appendix 2) and video conferencing for debriefing - provided STUPS with practical insights into the benefits (e.g., improved accessibility, reusability, and preparation efficiency) and limitations of digital solutions, particularly in group composition, IPC, error tracking, and structured debriefing. These experiences shaped initial development needs, further refined through discussions with instructors, participants, and stakeholders. In line with DSR Step 2, these needs were translated into concrete development objectives (see table 1 (Tab. 1)) to guide the creation of a digital, simulation-based application for training and debriefing SA in interprofessional teams.

The defined objectives reflect key functional and pedagogical requirements. A core goal was to create a realistic virtual training scene replicating a hazard-embedded patient room, aligned with the German Patient Safety Initiative’s competency catalogue and the Swiss Patient Safety Foundation’s* Interactive Learning in the Room of Horrors* guide [66], [59]. Within this scene, the high-pressure conditions of daily clinical work – requiring participants to operate under time constraints and accurately identify patient safety hazards – were to be simulated through gamification elements. To support IPC, a goal was to accommodate 30-40 participants, grouped into interprofessional teams of about five. These teams were to enter the virtual ROE simultaneously but operate independently, documenting identified hazards in a digital notebook shared within each team. To address the lack of integrated solutions combining training and debriefing, the main objective was to embed debriefing as an integral part of the training process. Accordingly, the application was aimed to support instructor-led debriefing with structured access to each team’s documentation, enabling review of identified hazards, discussion of solutions, and recognition of high-performing teams. Additional goals included scalability for varied use cases and high usability by avoiding specialized equipment (e.g., VR/AR), ensuring low-threshold implementation without sacrificing realism. 

Following DSR, these objectives guided the web-based software development (step 3). The prototype was demonstrated (step 4) in a competitive classroom challenge, outperforming four other solutions, and subsequently tested to assess the justification for further development into a fully functional application.

## 4. Results

### 4.1. Piloting the prototypical application

An evaluation tested the prototype’s real-world applicability, user experience, and development potential. It was conducted during a training session with five final-year medical students and nine Health and Nursing Science students (n=14), organized into three interprofessional teams. The design followed the Kirkpatrick model, which evaluates training across four levels: reaction, learning, behaviour, and results [67], [68], [69], [70], [71]. To operationalize these, a post-training survey combined Likert-scale items on perceived learning, relevance, difficulty, and interprofessional exchange with open-ended prompts on key experiences, memorable errors, and overall impressions (see attachment 1 , appendices 3 and 4).

The results (see attachment 1 , appendix 5) indicate that the prototype was well received and met its exploratory aims. All participants reported benefiting from interprofessional exchange, and 86% found the embedded hazards relevant to their professional context. Over two-thirds strongly agreed the training was educational, and most would recommend the format. Qualitative responses supported these findings, emphasizing the value of teamwork under time pressure and the importance of discussing patient safety from multiple perspectives. Several participants endorsed continued development and offered constructive suggestions. Overall, the evaluation confirmed the prototype’s viability and justified further development. Insights gained directly informed subsequent development and contributed to the refined application presented in the next section.

### 4.2. Developed digital, simulation-based application 

Building on insights from the pilot testing of the prototype, the application was refined into a fully functional, web-based platform. This section presents the final version: a simulation-based application designed to train interprofessional teams in SA, integrating a realistic virtual environment, collaborative tools, performance analytics, and structured debriefing.

#### 4.2.1. User-centric design

**Figure 2:**
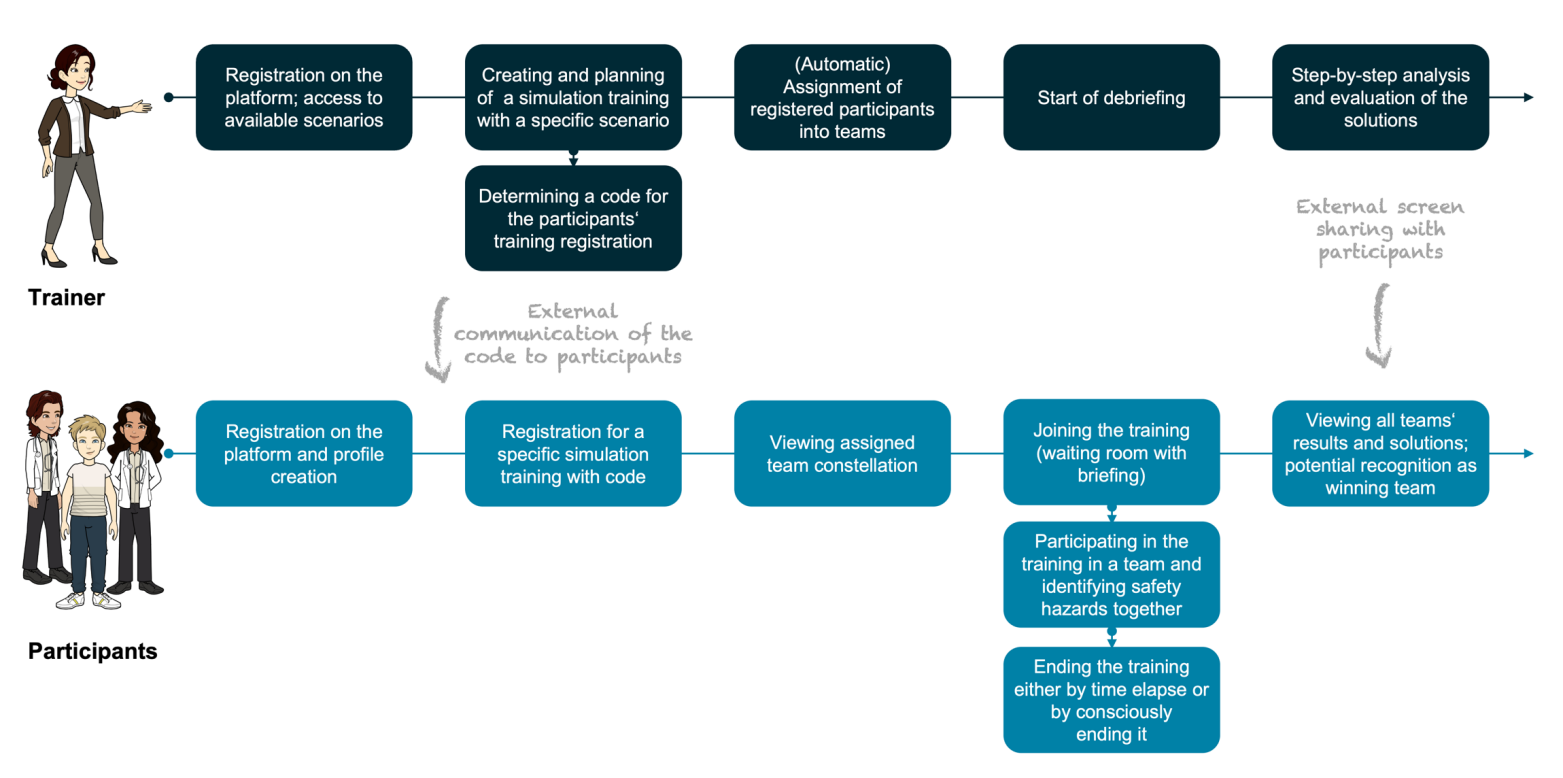
User-centric platform

The application features a platform-based design tailored to two user groups: trainers and participants (see figure 2 (Fig. 2)). For *trainers*, the platform serves as a centralized hub for planning and conducting training sessions and debriefings. Trainers register, access virtual scenarios, and use the dashboard to create and schedule simulations (see attachment 1 , appendix 6). They assign participants to groups, either automatically or manually, via a unique registration code shared by email or QR code. This simplifies coordination, supports digital collaboration and reduces administrative effort.

*Participants* register, create profiles (see attachment 1 , appendix 7), and use the code to enrol in sessions, with automated assignment into an interprofessional team visible in their profiles (see attachment 1 , appendix 8). Participants enter a virtual waiting room, receive a briefing, and engage in the training session, concluding by set time or team decision (see attachment 1 , appendix 9). The application assumes a video-conference setup for communication, using external interfaces for sharing registration codes and debriefing results. Verbal communication during training is crucial, and trainers initiate post-training debriefings with a single click (see attachment 1 , appendix 10), allowing for step-by-step analysis and evaluation.

#### 4.2.2. Virtual training scene and gaming character

The application simulates the ROE using 360-degree, high-resolution images. Participants enter a dynamic scene with team members, a central timer, and interactive elements like a notebook and patient file (see attachment 1 , appendix 11). The room features 13 errors linked to patient safety hazards (see attachment 1 , appendix 12). Grey-shaded interactive elements allow detailed examination, such as the patient’s side table (see attachment 1 , appendix 13). Selecting an element triggers a pop-up for participants to describe hazards, with inputs recorded in the notebook. The simulation mimics a high-pressure environment, with a red-flashing countdown in the final minute to heighten stress.

#### 4.2.3. Interprofessional teams and interactive collaboration

**Figure 3:**
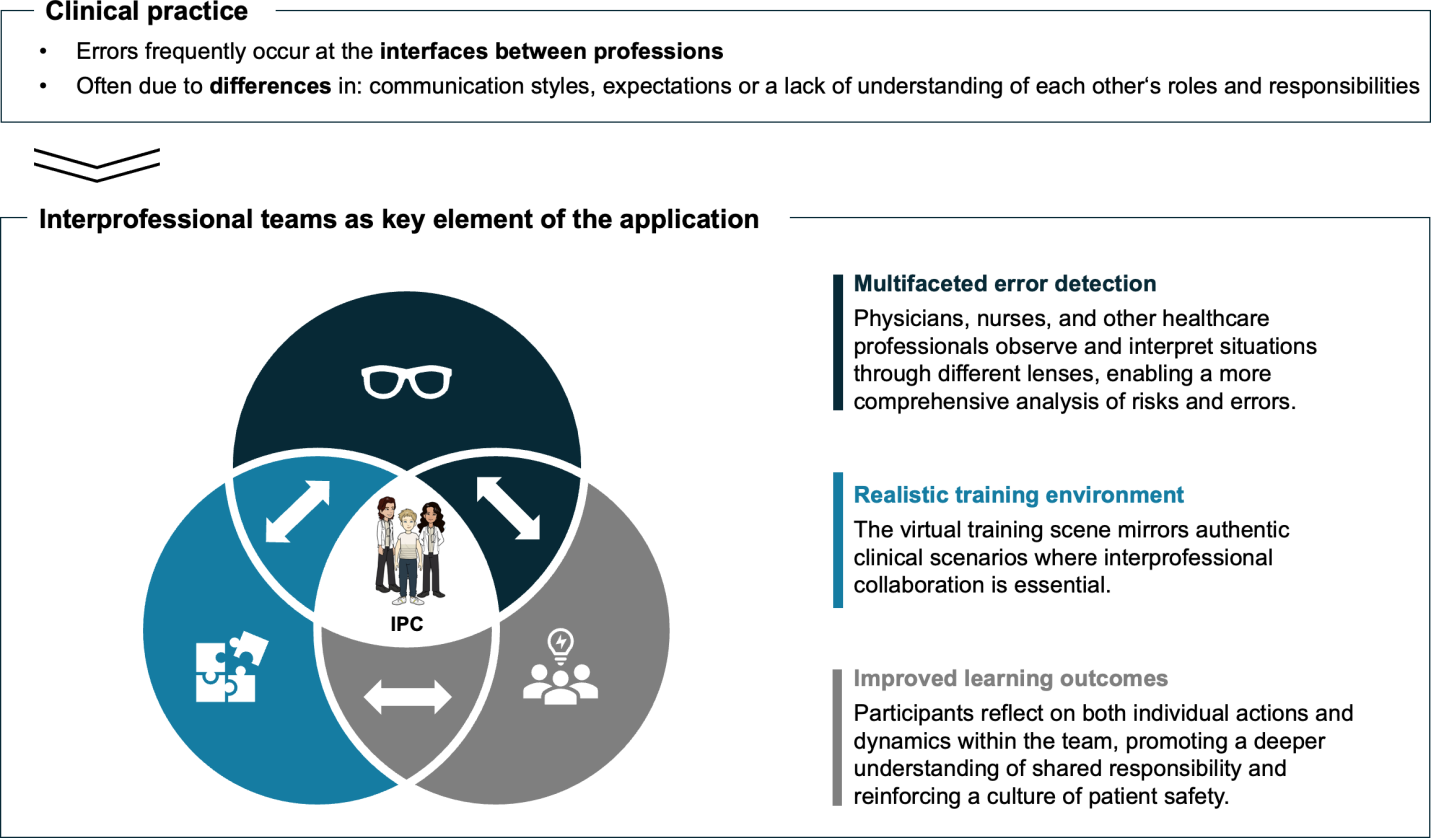
Relevance of interprofessional collaboration

The application supports scalable IPC, accommodating many participants across multiple teams. Trainers can assign participants via the dashboard, using automatic or manual team assignment into professional teams with a drag-and-drop interface (see attachment 1 , appendix 14, 15). This ensures an optimal professional mix and reduces trainers' administrative burden. Participants can immediately see their assigned teams, including names and backgrounds (see attachment 1 , appendix 8). Collaboration is further supported by a shared digital notebook for documenting and discussing errors (see attachment 1 , appendix 16), with notifications for real-time collaboration. All in all, interprofessional teams are central to the ROE, enabling realistic collaboration and richer hazard detection across professional boundaries (see figure 3 (Fig. 3)).

#### 4.2.4. Analytics and evaluation

The application monitors two key metrics: the time teams spend in the simulation and their performance in identifying and documenting errors. These metrics are vital for debriefing, fostering interprofessional dialogue, and helping trainers to discuss errors that may have gone unnoticed, evaluate outcomes and recognize top teams. By automatically tracking errors identified by each group and linking them to specific teams, the application enables transparent performance monitoring. Additionally, all results are stored systematically, allowing for long-term analysis to continuously enhance training and explore how risks are perceived across professional groups.

#### 4.2.5. Debriefing

**Figure 4:**
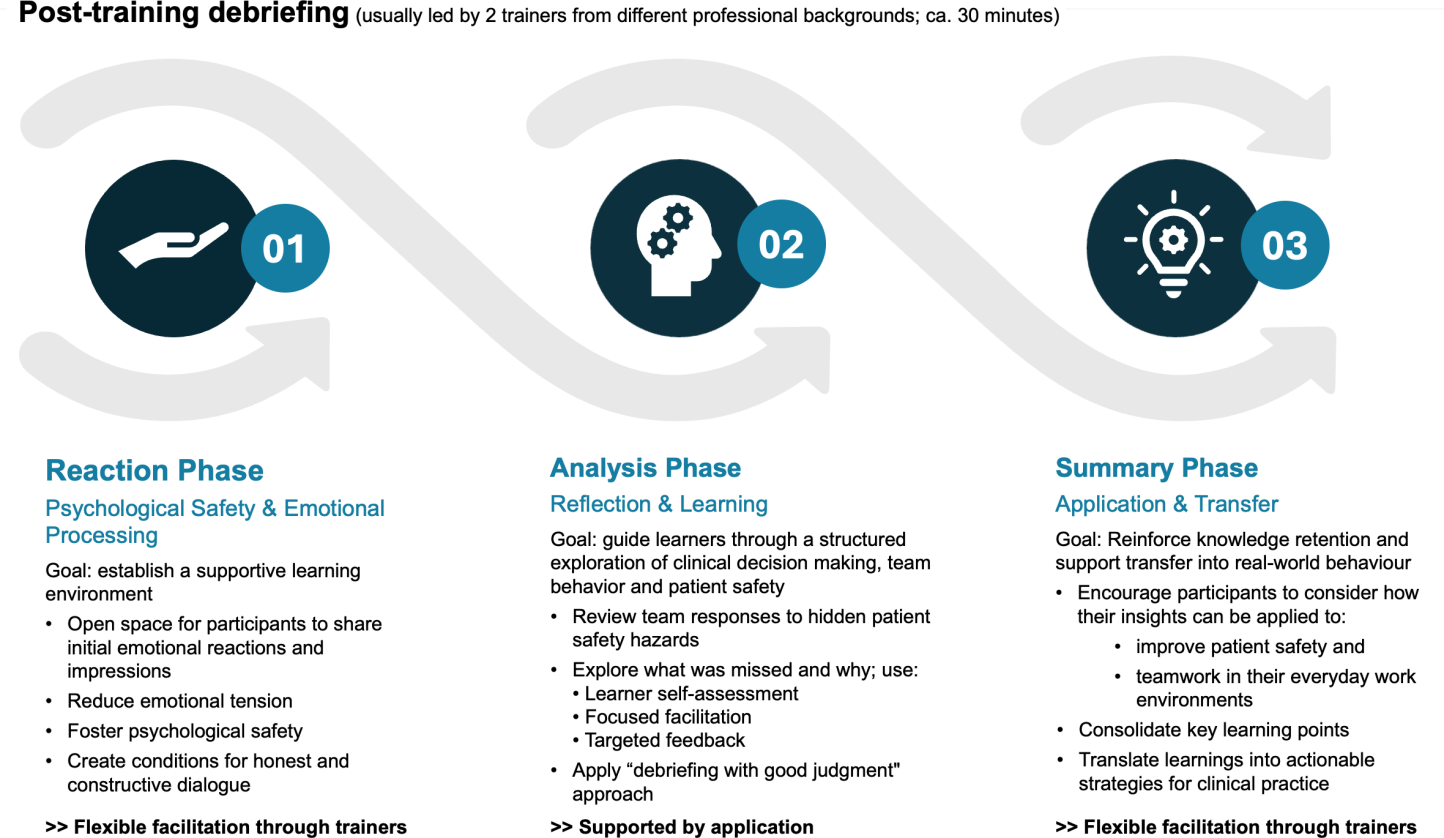
Debriefing process

After the training session, debriefing is launched via the trainer’s dashboard (see attachment 1 , appendix 10). The process is structured around the DASH framework [72] and enriched by PEARLS strategies [73] to foster psychological safety and guide reflection across three phases: reaction, analysis, and transfer. The application supports the analysis phase through a structured review of team responses, model solutions, and performance feedback, while instructors flexibly guide the other phases. Flexible facilitation enables both “debrief-to-learn” and “debrief-to-manage” approaches [40], in line with recommendations for post-training reflection to improve clinical reasoning and patient safety [48]. For an overview, see figure 4 (Fig. 4).

## 5. Discussion

The defined development objectives were successfully realized in a fully operational, web-based application. The final version demonstrates the feasibility of delivering a realistic, collaborative, and scalable simulation experience for interprofessional teams without requiring specialized equipment:

- A high-resolution, 360-degree virtual patient room replicates a clinical environment in which embedded patient safety hazards must be identified, marked, and described collaboratively by participants within an interprofessional team.
- Gamification, including time constraints and team-based competition, introduces pressure and engagement mechanisms intended to simulate aspects of clinical urgency and support the training of SA under realistic conditions.
- IPC is supported through scalable participant management with automated or manual team assignment. A shared digital notebook enables transparent collaboration, while cross-group debriefings further promote interprofessional exchange and shared learning.
- Integrated analytics track time and error identification accuracy, supporting structured, data-informed debriefings and reducing instructors’ workload.
- Debriefing is embedded into the training process, offering guided access to team documentation and enabling flexible, instructor-led reflection.
- The browser-based architecture ensures easy usability and low-barrier access, enabling location-independent training without the need for physical rooms.
- The modular application architecture allows for scenario expansion and complexity scaling.

Despite these achievements, limitations remain. First, although informed by prototype evaluation, the final version has not yet undergone formal testing. Its impact on SA and IPC remains to be empirically validated. Second, while theoretically grounded, no empirical link has been established between application use and SA improvement. This reflects broader challenges in measuring SA in team-based contexts. Existing tools such as SA Global Assessment Technique or Team SA [27], [74], [75] may serve as starting points but often yield ambiguous results in team settings [5]. The complexity of interprofessional dynamics and diverse knowledge contributions complicates SA assessment and calls for systems-level approaches like distributed situation awareness [76], [77], [78]. Future efforts should therefore focus on adapting or combining such methods to assess whether the application measurably improves SA. Lastly, while the application was designed for efficiency, cost-effectiveness, reusability, and scalability, these remain implementation prospects to be explored through broader deployment and longitudinal studies.

## 6. Conclusion

This report addressed the question of how interprofessional teams can be virtually trained and debriefed for SA to mitigate patient safety hazards. Guided by DSR, we developed a digital, simulation-based application that combines SA training in a virtual ROE with structured, instructor-led debriefing. The application aligns with global trends in medical education that emphasize simulation to strengthen teamwork, communication, and error management [23], [5], [79], [80], [81]. By integrating IPC and reflective debriefing, it supports WHO recommendations and educational research on team-based learning, human factors, and psychologically safe feedback [5], [9], [38], [48], [49], [82], [83]. Overall, the development of the application contributes to the growing role of simulation in interprofessional education and learning from error to prevent harm [38], [83]. While the feasibility of the application has been demonstrated, its educational efficacy – particularly its impact on SA and IPC – requires further empirical validation.

## Abbreviations

- DSR: Design Science Research
- IPC: Interprofessional Collaboration
- NTS: Non-technical Skills
- ROE: Room of Error
- SA: Situational Awareness
- STUPS: Stuttgarter Pädiatrie- und Patientensimulator

## Acknowledgements

We extend our sincere gratitude to Prof. Dr. Kai Holzweißig, Dean of Studies and Program Director of Business Informatics at DHBW Stuttgart, for his significant role as initiator and supporter of the project. His openness to innovative teaching approaches greatly contributed to the project's realization. We also wish to acknowledge Daniel Seger, a student of Business Informatics Data Science at the DHBW Stuttgart, for his invaluable technical contributions to this project, which have been vital to the project’s success. Daniel's enduring motivation and unwavering commitment have been instrumental in creating the first and second artifact, as well as in the long-term development and optimization of the application. 

## Notes

### Data privacy and ethical considerations 

Innovative teaching formats may be evaluated under the legal framework of experimentation in higher education (LHG BW §32). The pilot of the Virtual Room of Error was designed and conducted in accordance with ethical principles, including voluntary participation, informed consent, and data protection in compliance with the GDPR.

### Authors’ ORCIDs

- Veronika Spielmann: [0009-0003-9428-8075]
- Margrit Ebinger: [0009-0007-3378-3258]
- Christina Jaki: 80009-0002-0797-5452]

## Competing interests

The authors declare that they have no competing interests. 

## Supplementary Material

Supplementary material

## Figures and Tables

**Table 1:**
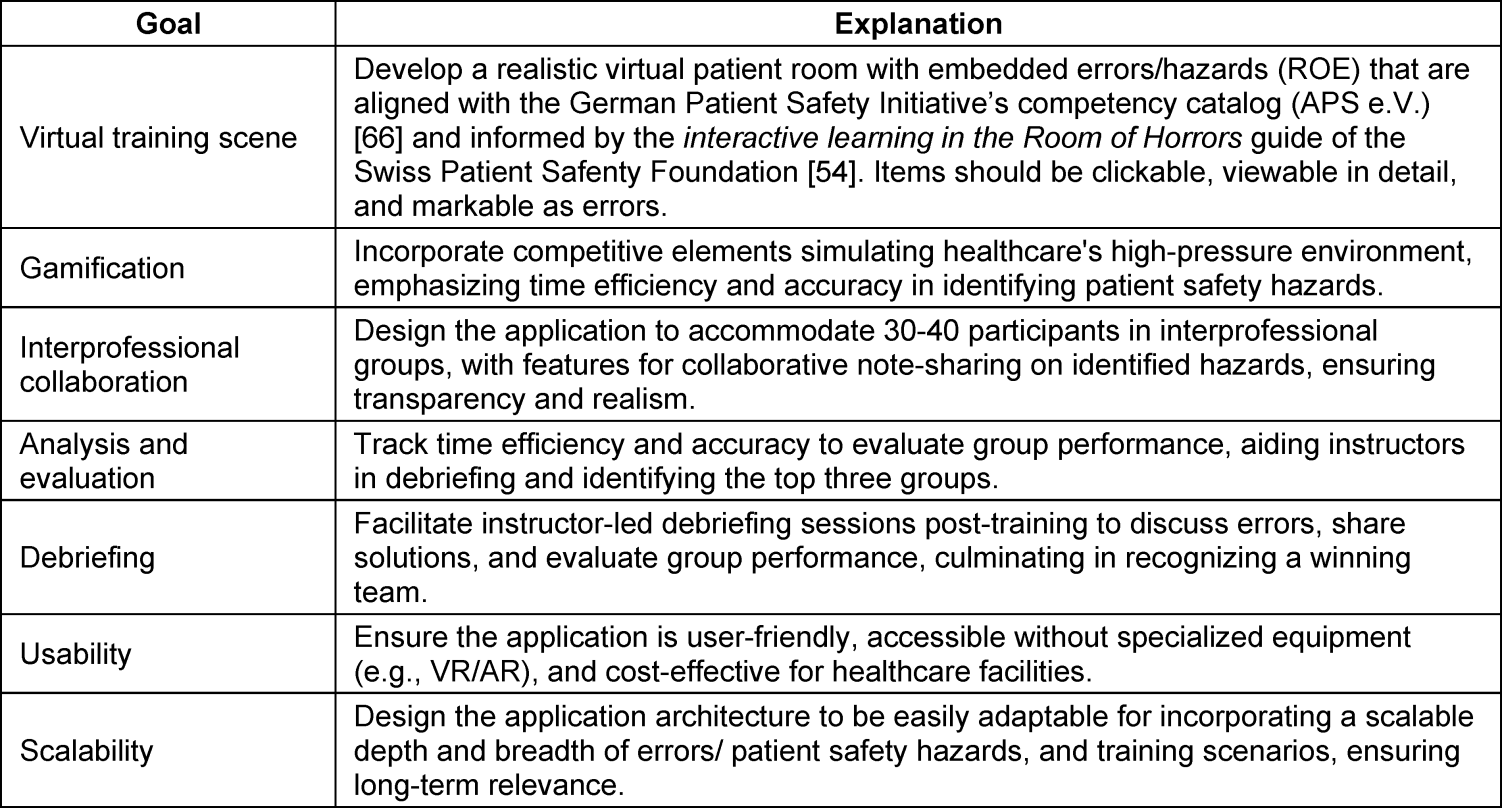
Objectives guiding application development
