# Supplementary material for: Development of a digital application to train and debrief situational awareness in interprofessional teams: A simulation-based approach to mitigate patient safety hazards with a virtual room of error
Source: GMS J Med Educ. 2026 Feb 17;43(2):Doc24. doi: 10.3205/zma001818 (PMC12936916; doi:10.3205/zma001818)
Supplement: Supplementary material [file JME-43-24-s-001.pdf]

Attachment 1: Supplementary material

Appendix 1: STUPS Facilities and “Room of Error” 2

Appendix 2: Digital "Room of Error" 3

Appendix 3: Evaluation Survey 4

Appendix 4: Design of the Evaluation Survey 5

Appendix 5: Results of the Evaluation Survey 5

Appendix 6: Application - Trainer's view: planning of training 10

Appendix 7: Application - Registration on platform and profile creation 11

Appendix 8: Application - Participant's view: team members 12

Appendix 9: Application - Participant's view: waiting room 15

Appendix 10: Application - Trainer's view: debriefing session 16

Appendix 11: Application - Virtual scene and 360-degree view 20

Appendix 12: List of patient safety hazard in the STUPS "Room of Error" 22

Appendix 13: Application - Virtual scene and clickable error element 23

Appendix 14: Application - Trainer's view: administer teams 25

Appendix 15: Application - Trainer's view: team assignment and additional teams 26

Appendix 16: Application - Virtual scene and notification alert 28

Appendix 1: STUPS Facilities and “Room of Error”

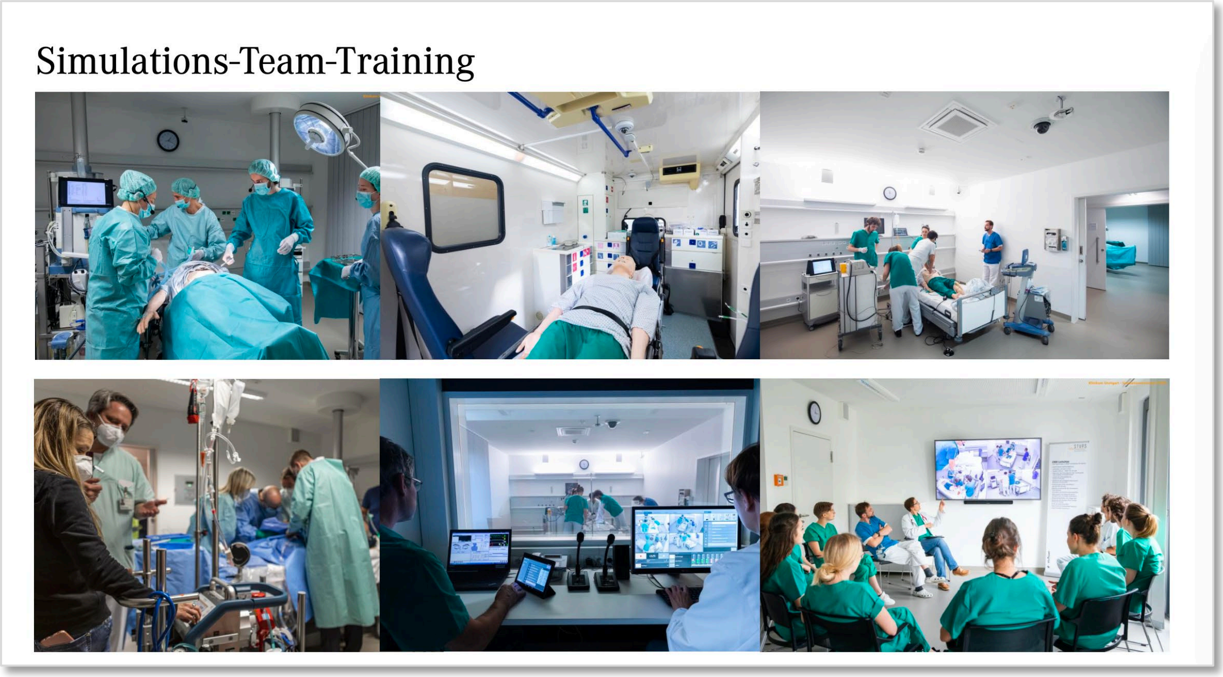

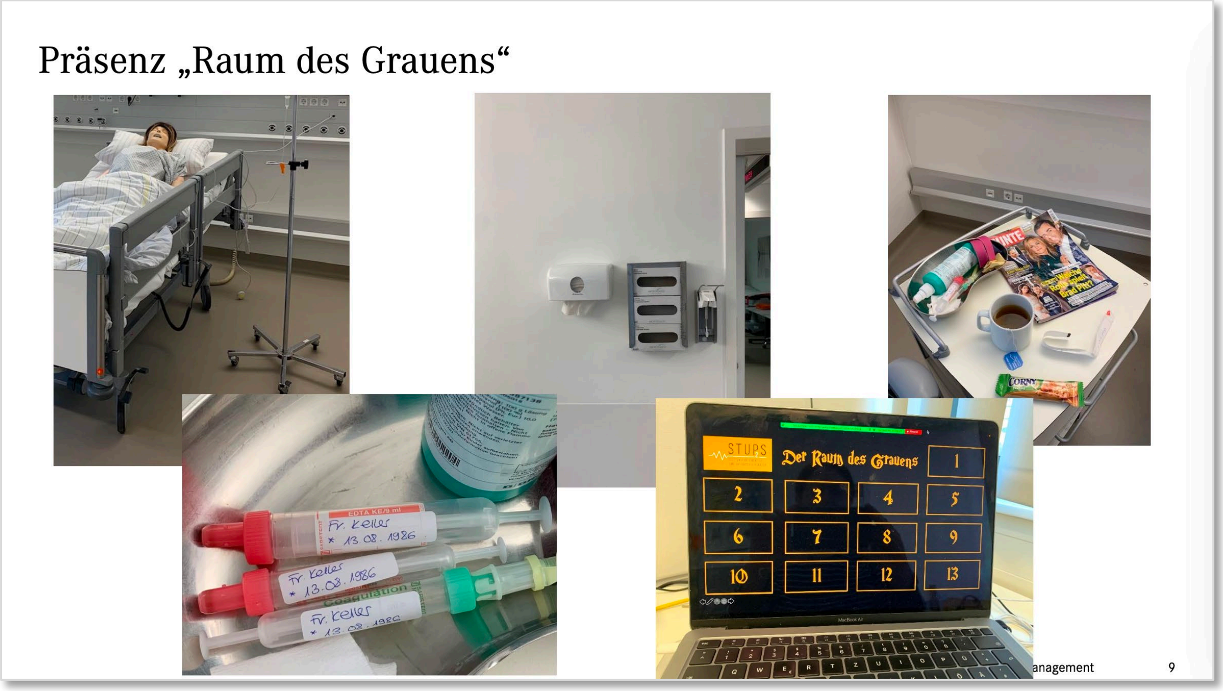

Appendix 2: Digital "Room of Error"

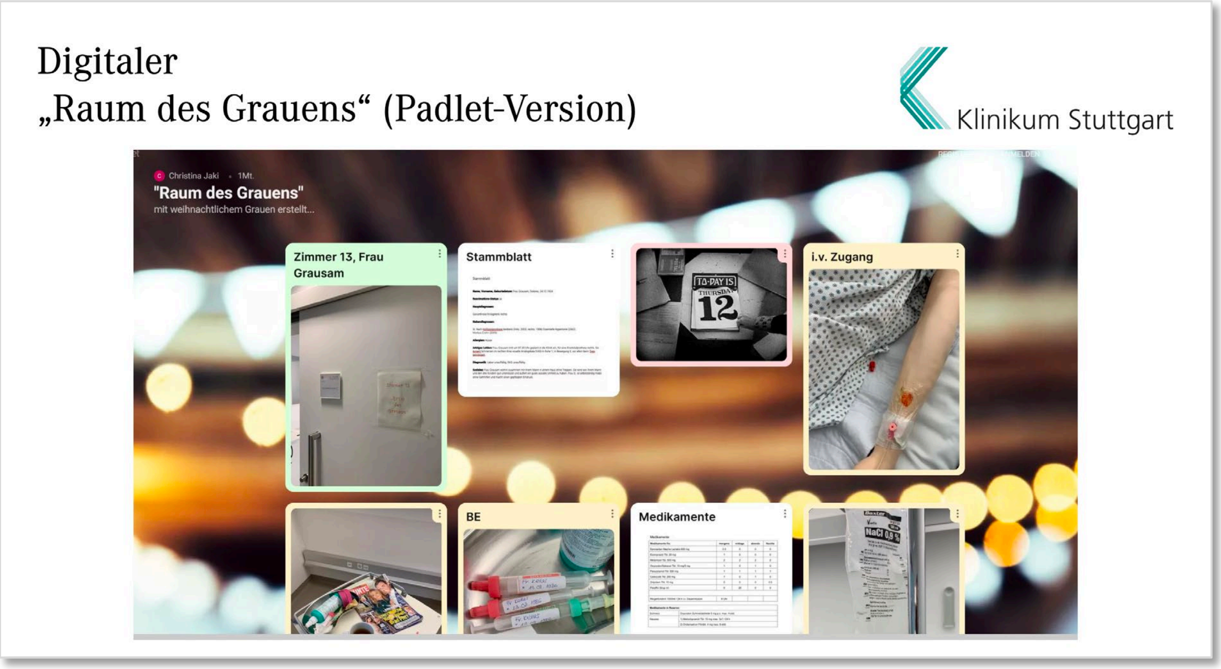

Attachment 1 to Spielmann V, Ebinger M, Jaki C. *Development of a digital application to train and debrief situational awareness in interprofessional teams: A simulation-based approach to mitigate patient safety hazards with a virtual room of error.* GMS J Med Educ. 2026;43(2):Doc24. DOI: 10.3205/zma001818

Appendix 3: Evaluation Survey

Nr.	Survey Question	Question type
1.	I am<br>☐ a doctor in the practical year<br>☐ an undergraduate student in Applied Health and Nursing Sciences	Dichotomous question
2.	I have benefited from the exchange with the other professional group	Forced choice Likert scale (level of agreement from 1 = ++ to 4 = --)
3.	The hidden errors and dangers are relevant to my everyday professional life.	Forced choice Likert scale (level of agreement from 1 = ++ to 4 = --)
4.	It was difficult to find the errors and patient safety hazards.	Forced choice Likert scale (level of agreement from 1 = ++ to 4 = --)
5.	I found the "Room of Horrors" educational.	Forced choice Likert scale (level of agreement from 1 = ++ to 4 = --)
6.	I would recommend the "Room of Horrors."	Free text, qualitative answer
7.	The "most exciting" patient safety hazard for me was:	Free text, qualitative answer
8.	… because:	Free text, qualitative answer
9.	My most important experience in the "Room of Horrors" was:	Free text, qualitative answer
10.	What else I would like to say:	Free text, qualitative answer

Appendix 4: Design of the Evaluation Survey

Level according to Kirkpatrick model	Evaluation goal	Survey questions
1. Reaction	assessing the immediate response to the training program in terms of feelings about the training and learning experience	4, 10
2. Learning	finding out actual learnings from the training, for example in terms of knowledge, skills, and attitudes.	2, 5
3. Behaviour	evaluating the transfer of learning to the workplace relating to changes in behaviour and the implementation of learning in the real-world	3, 9
4. Results	assessing the overall impact of the training on initial goals and objectives	6, 7, 8

Appendix 5: Results of the Evaluation Survey

n=14

Distributions of Professions:

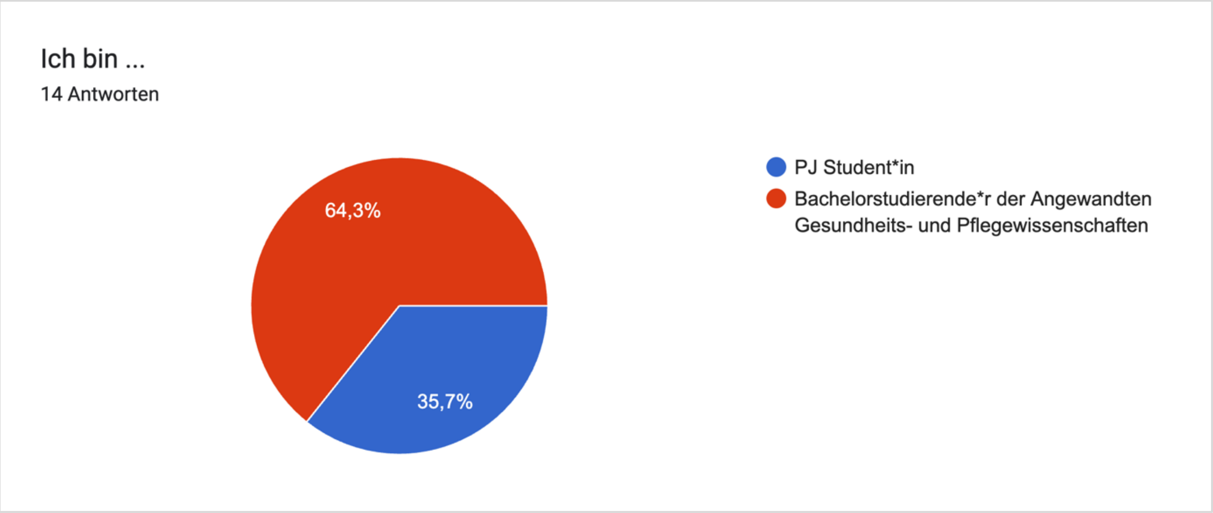

Perceived benefit of interprofessional exchange:
(Evaluation level: learning)

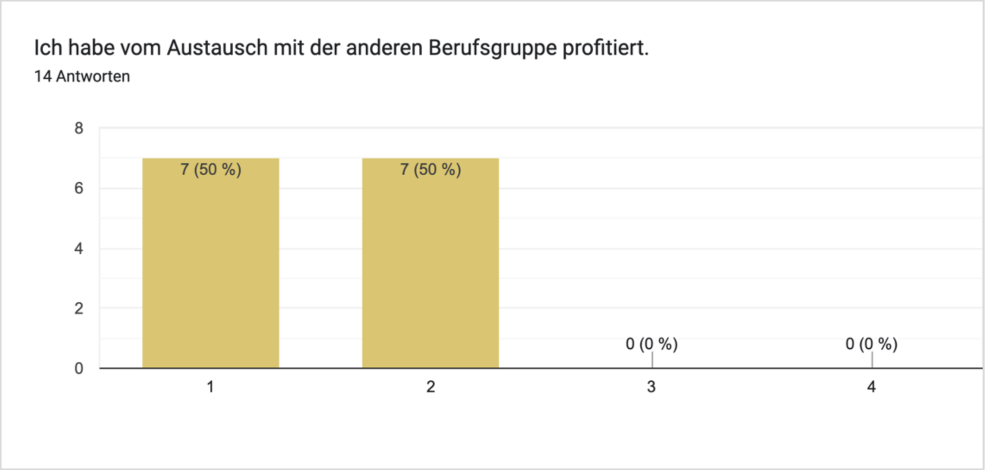

Perceived relevance of errors and hazards:
(Evaluation level: behavior)

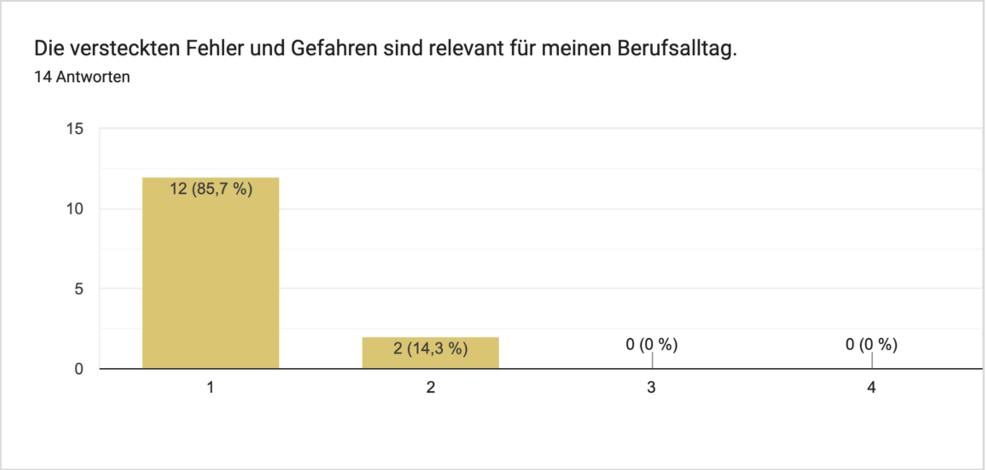

Perceived difficulty of the identification of errors:
(Evaluation level: reaction)

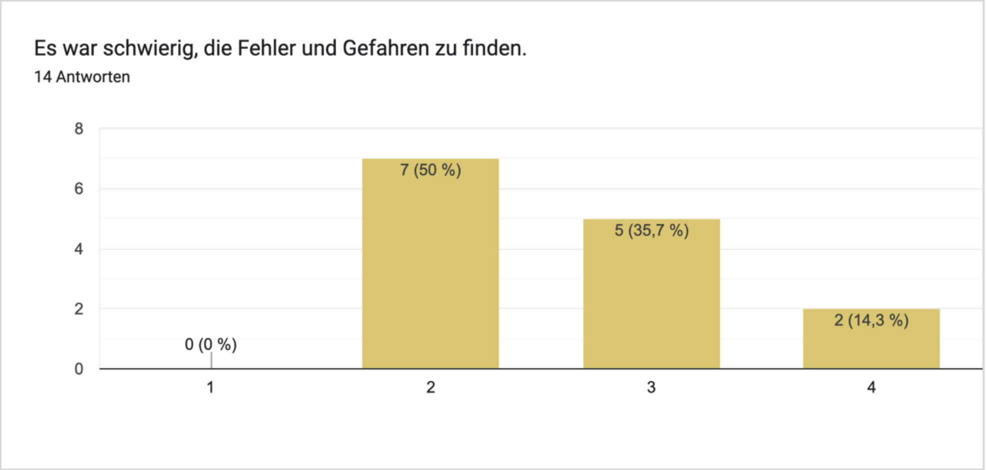

Perceived educational value:
(Evaluation level: learning)

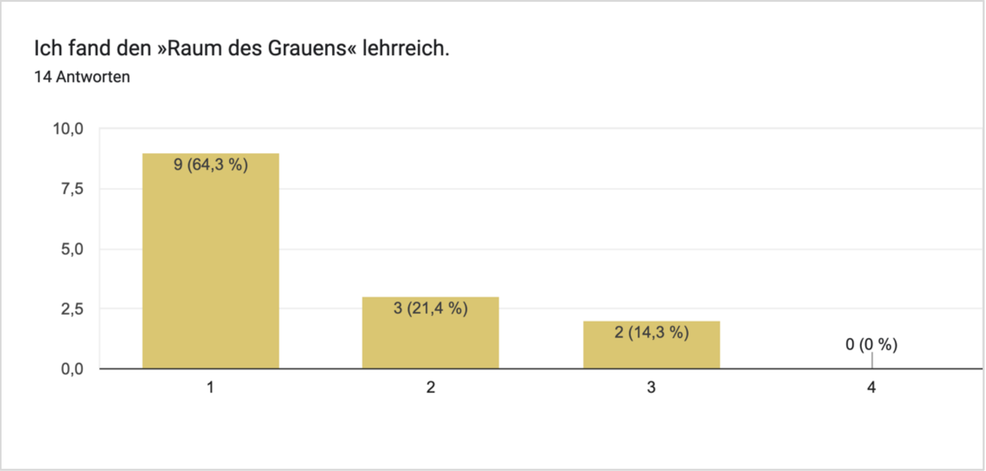

Training endorsement:
(Evaluation level: results)

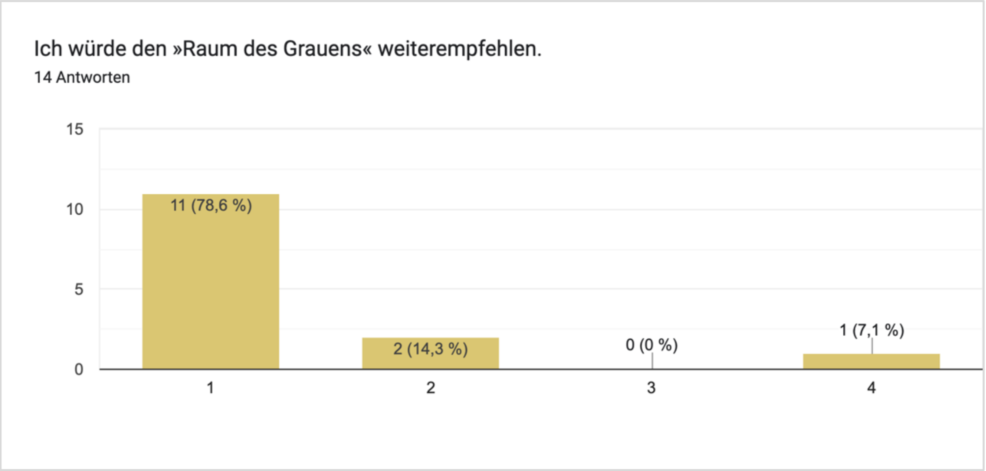

Most “exciting” patient safety hazard:
(Evaluation level: results)

Der "spannendste" Fehler für mich war...
10 Antworten

- Namensverwechslung
- Fehlende Thromboseprophylaxe
- Fehler in der Pat.akte
- Patientenverwechslung bei Blutröhrchen
- Informationen & Verordnungen in Patientinnenakte, die nicht berücksichtigt wurden
- Falsches Bein markiert
- Thromboseprophylaxe
- Falsche Name
- Die Akte

weil...
9 Antworten

- es einer der gravierendsten Fehler sein kann
- Man denk nicht dran
- diese unerwartet waren
- leider Realität auf Station und viele vor der Blutabnahme die Patientendaten nicht nochmal abfragen - kann vor allem bei Abnahmen für Blutprodukte lebensgefährlich werde, wenn eine Transfusionsreaktion entsteht.
- diese Fehler vehemente Auswirkungen haben.
- fataler Fehler
- Kann zu gefährliche Fehlern führen
- es ins Detail geht
- Da sie einige Fehler enthielt, die nicht direkt offensichtlich waren

Most important experience:
(Evaluation level: behaviour)

Meine wichtigste Erfahrung im »Raum des Grauens« war...

7 Antworten

- Dinge, wie dass das Bettgitter unten war, auf die man ansonsten immer achtet, sind mir nicht aufgefallen
- noch mehr auf Kleinigkeiten achten
- Zusammenarbeit
- Durch Teamarbeit konnten einige Fehler identifiziert werden. Alleine hätten nicht alle Fehler gefunden werden können.
- Bei Zeitdruck ist Teamarbeit lebensnotwendig
- Der Austausch
- Der Austausch miteinander über die Fehlerquellen

Other reactions and comments:
(Evaluation level: reaction)

Und was ich sonst noch sagen will...

9 Antworten

- sehr spannend und lehrreich
- Ich weiß nicht ob vorab auf die patientenkurve hingewiesen wurde, evtl das vor dem spielbeginn sagen
- war supertoll, jederzeit wieder gern!
- Toll gemacht!
- Vielen Dank für die Arbeit und die Bemühungen bei der Erstellung! Dieses Konzept sollte weitergeführt werden :)
- Gerne nochmal/regelmäßig
- War gespannt und Sehr gut undorganisiert
- Danke
- Die geschaffene kompetitive Atmosphäre durch das „Spiel" finde ich persönlich im Zusammenhang mit dem Grundgedanken wenig zielgerichtet. Ebenso waren die Erklärungen für die gefundenen Fehler und Bewertungen der abgegebenen Notizen nach der Meinung unseres Teams nicht kohärent. Insgesamt wäre es schöner gewesen wenn der Fokus auf der Zusammenarbeit und Kommunikation im Team gelegen hätte und nicht auf der ausschweifenden Auswertung der Angaben.

Appendix 6: Application - Trainer's view: planning of training

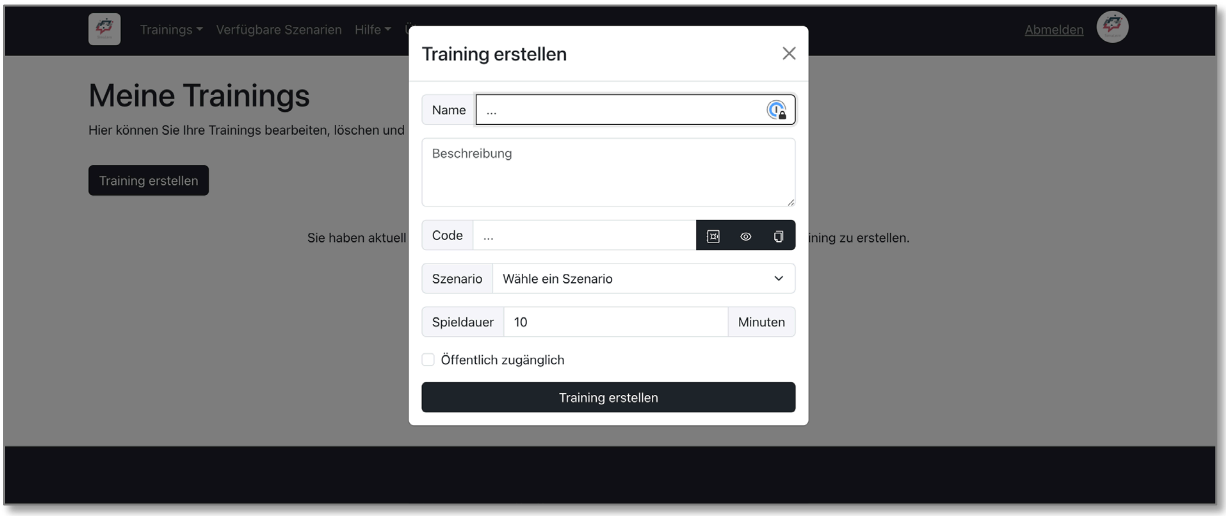

Interface for creating and planning a training.

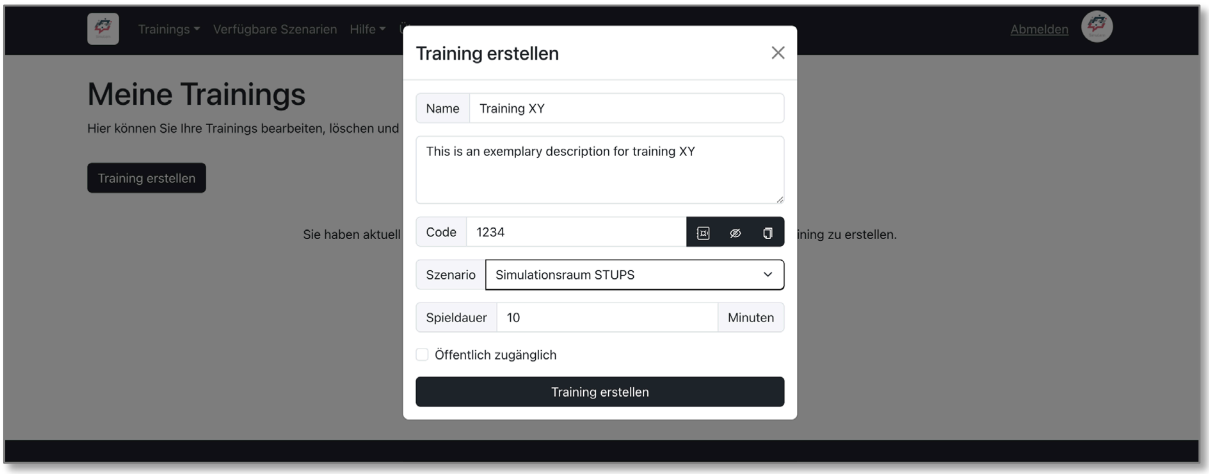

Exemplary filled interface.

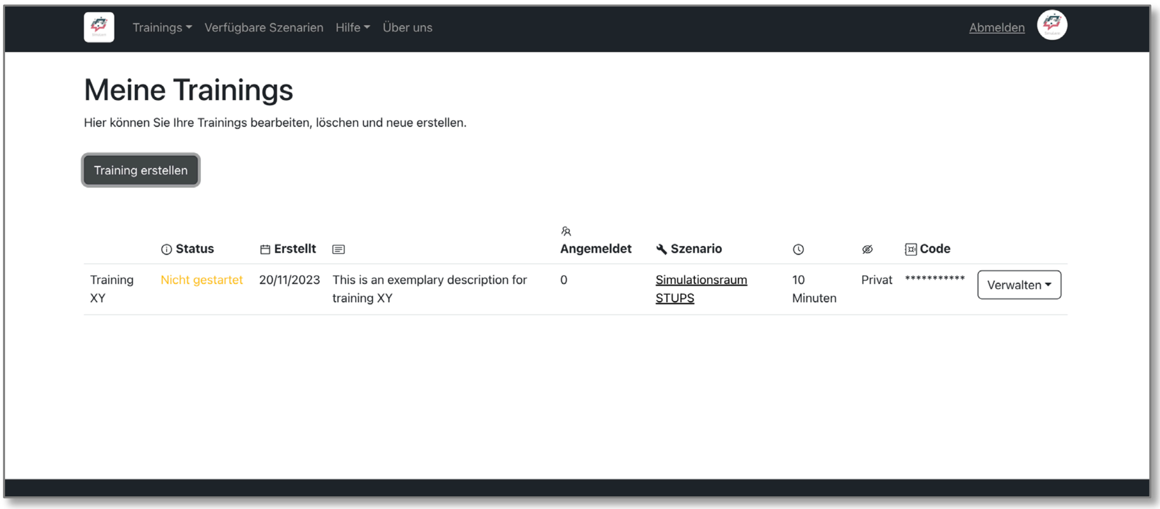

Overview of trainings in the trainer’s dashboard.

Appendix 7: Application - Registration on platform and profile creation

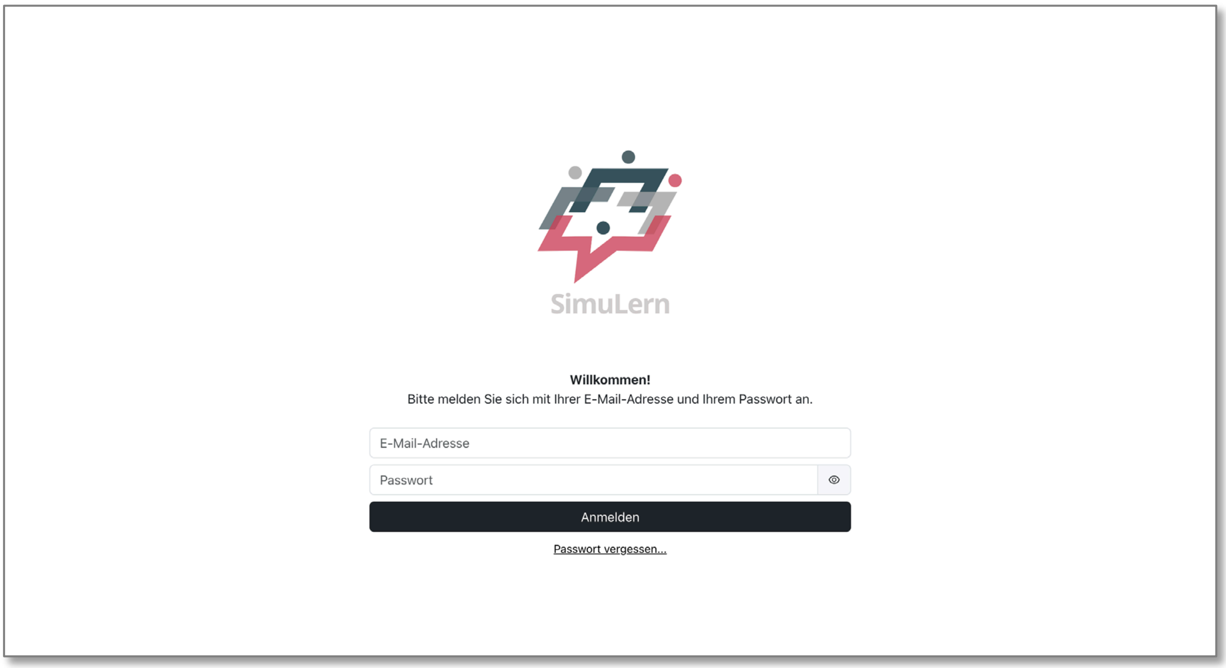

The login interface allows access to the application via email address and password.

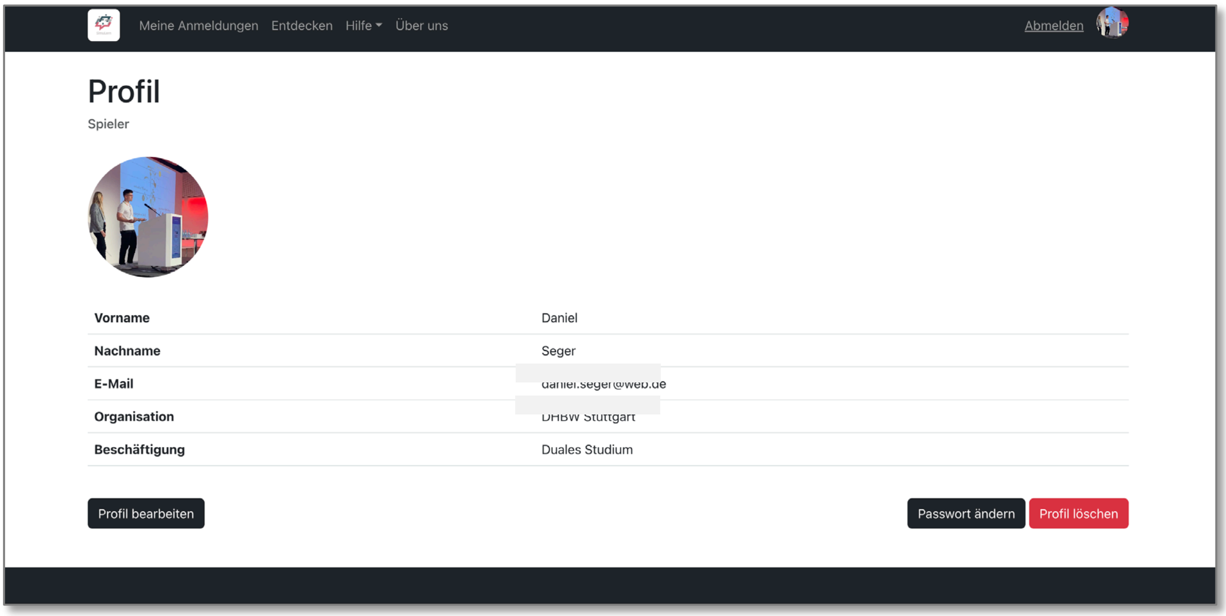

A user profile includes a picture, first name, last name, email address, organization, and profession. Users have the option to delete their profiles or change their passwords.

Appendix 8: Application - Participant's view: team members

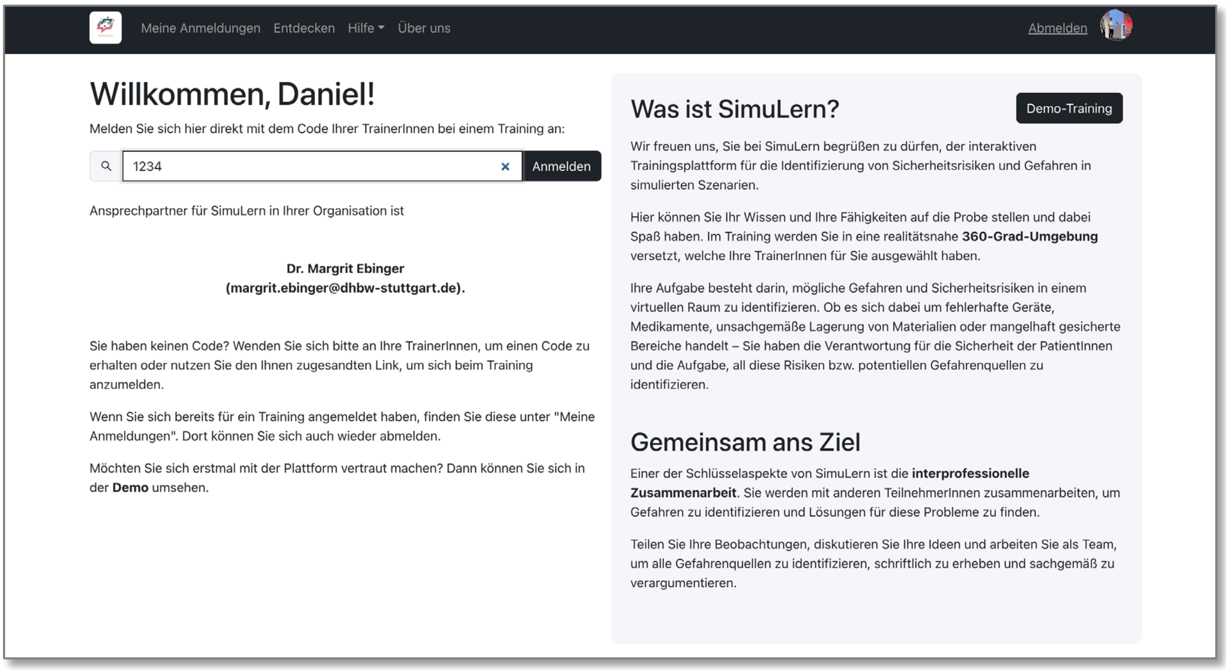

Participant’s dashboard view with the option to register for a training via code.

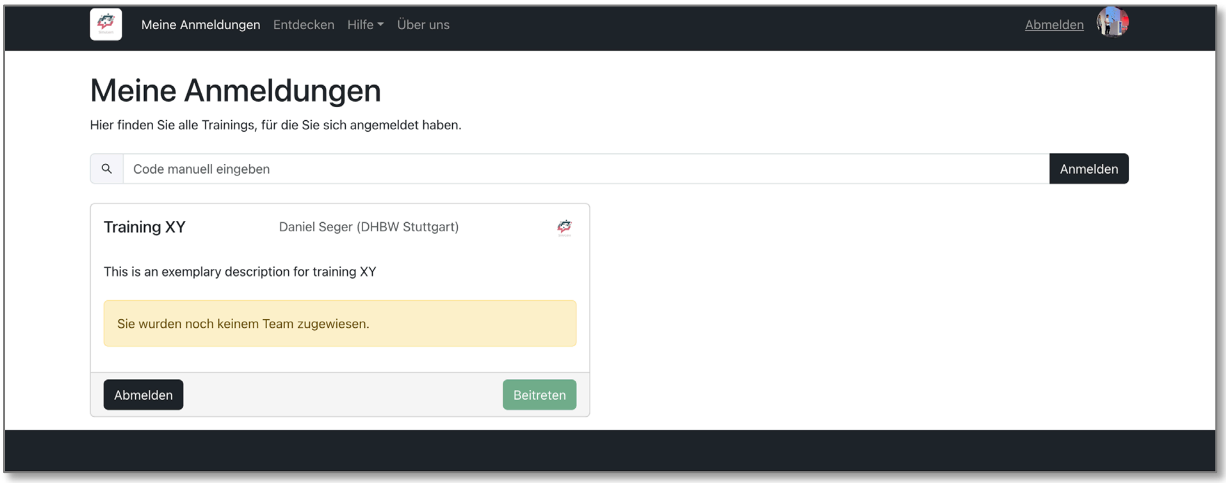

Participant's view after registration for a specific training via code and without being assigned to a team yet.

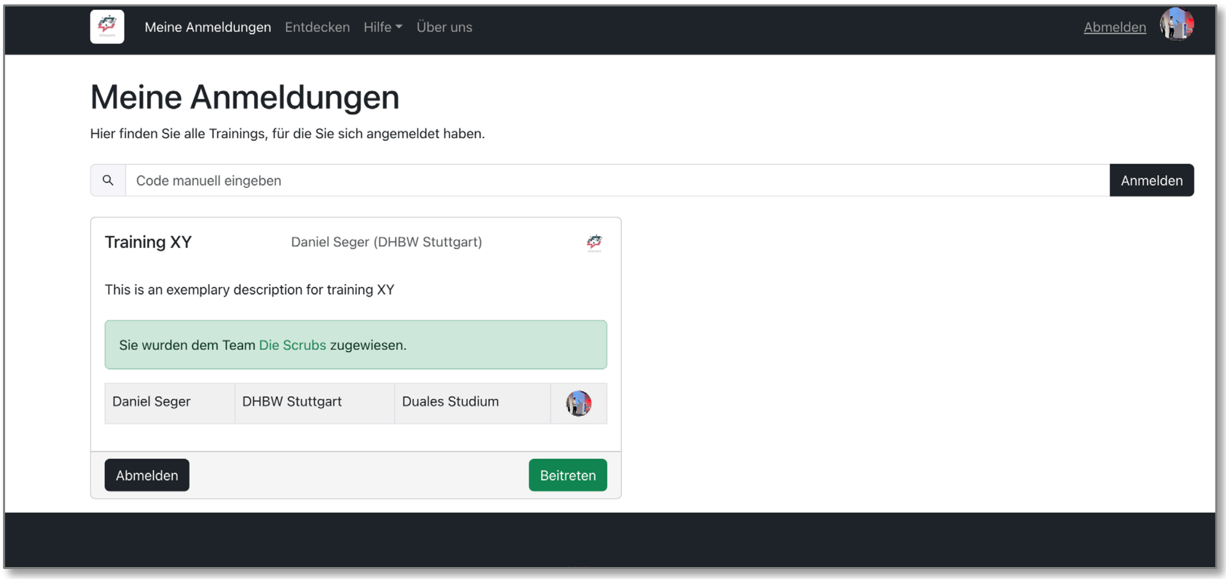

Participant's view (exemplary "Daniel Seger") as soon as the trainer assigned him to a team; by clicking "*Beitreten*" (join), the participant joins the virtual training.

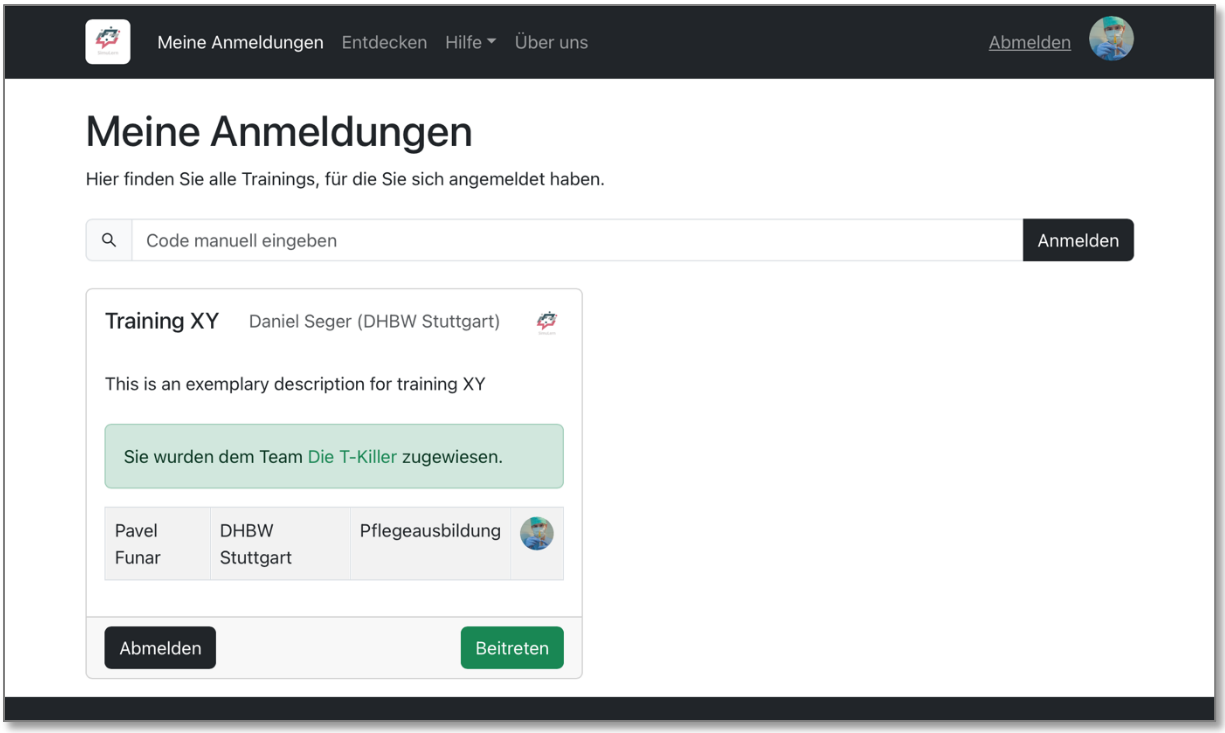

Participant’s view (exemplary “Pavel Funar”) as soon as the trainer assigned him to a team; by clicking “*Beitreten*” (join), the participant joins the virtual training.

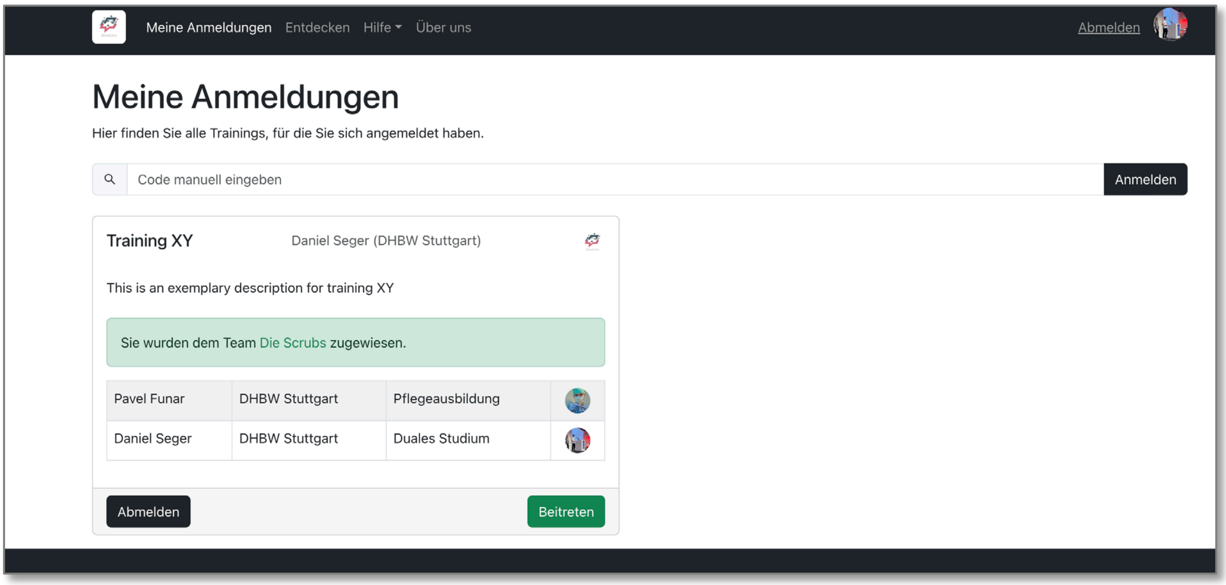

Participant’s view (exemplary “Daniel Seger”) as soon as the trainer assigned him to a team with another participant (exemplary “Pavel Funar”) that is his teammate. In this case, an interprofessional team was created by the trainer since “Daniel Seger” completes a “*Duales Studium*” (dual study) and “Pavel Funar” a “*Pflegeausbildung*” (nursing training).

Appendix 9: Application - Participant's view: waiting room

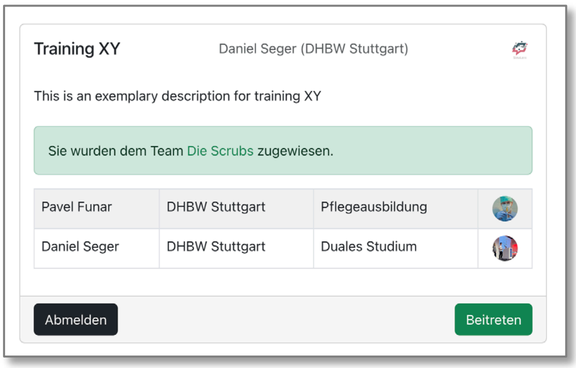

If a registered and assigned participant clicks "*Beitreten*" (join) and as long as the trainer has not started the simulation-based training yet, the participant will be put in a virtual waiting room where he / she can read more important information for briefing before the training.

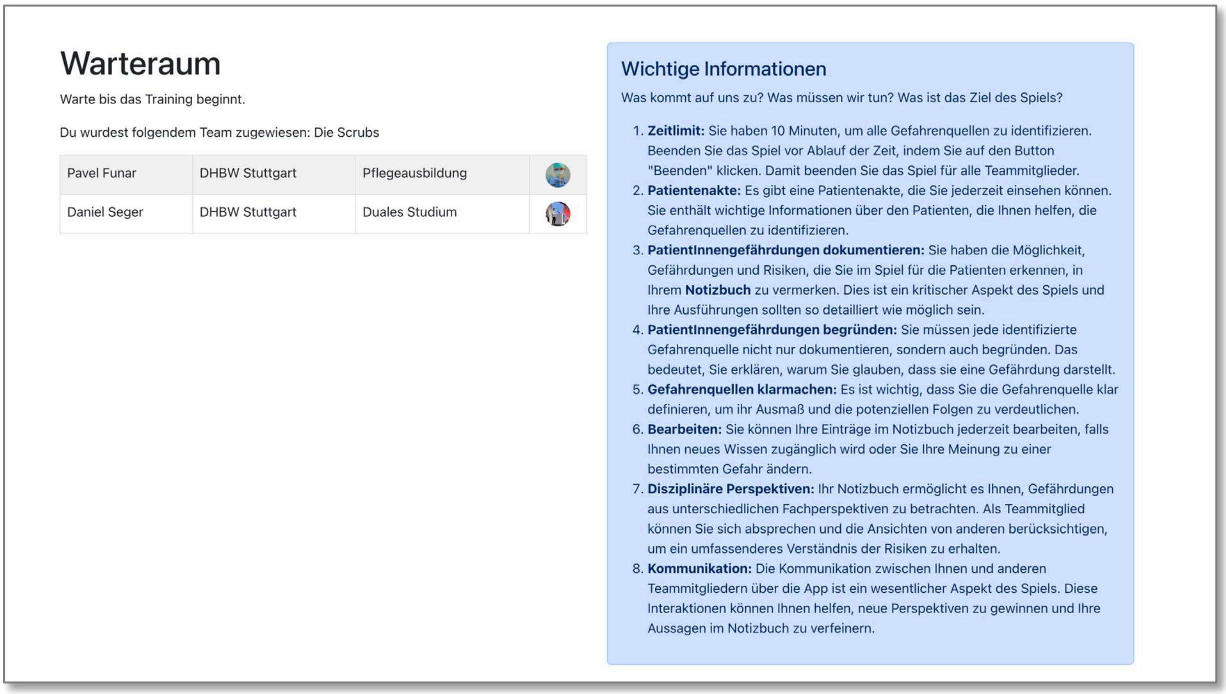

Appendix 10: Application - Trainer's view: debriefing session

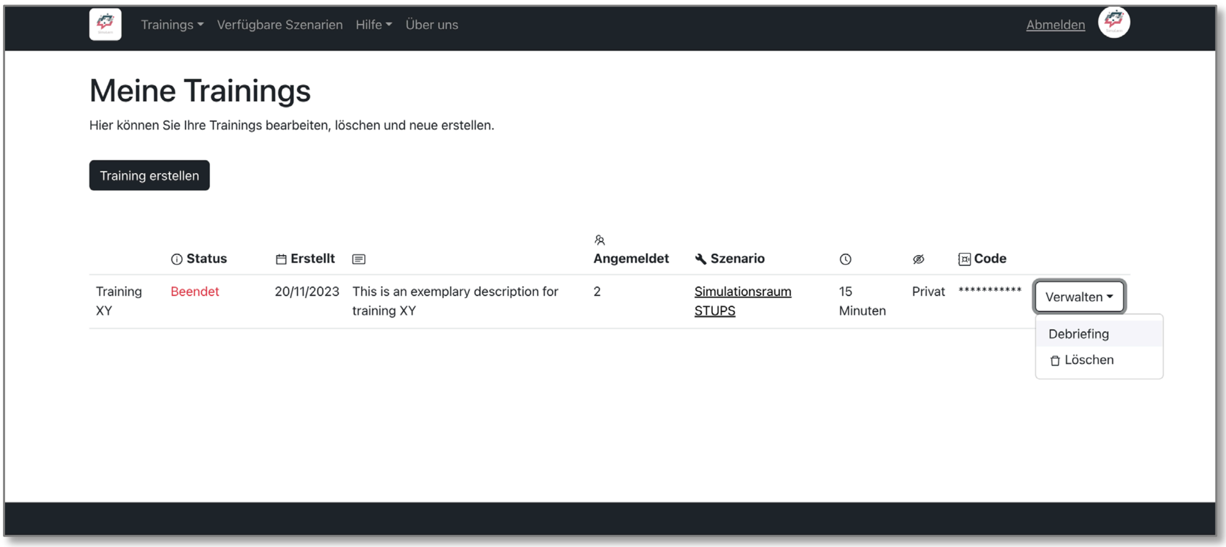

Once all teams have completed the training, the trainer can initiate the debriefing session by clicking *"Verwalten"* (administer) and then "Debriefing". This action will commence the step-by-step review of all errors and patient safety hazards.

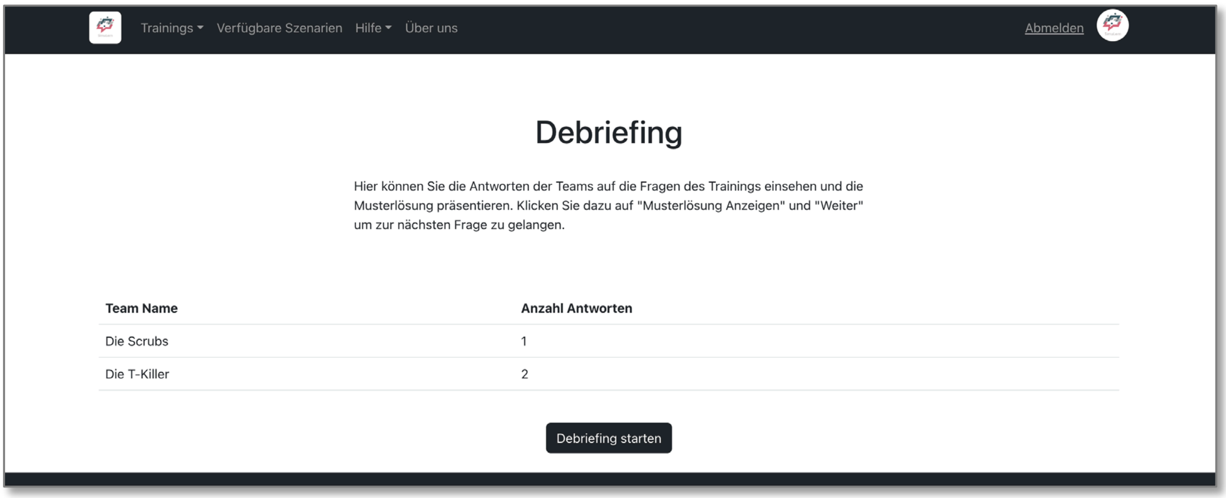

The debriefing begins with a summary showing all teams that participated in the specific simulation-based training, along with the number of answers each team provided.

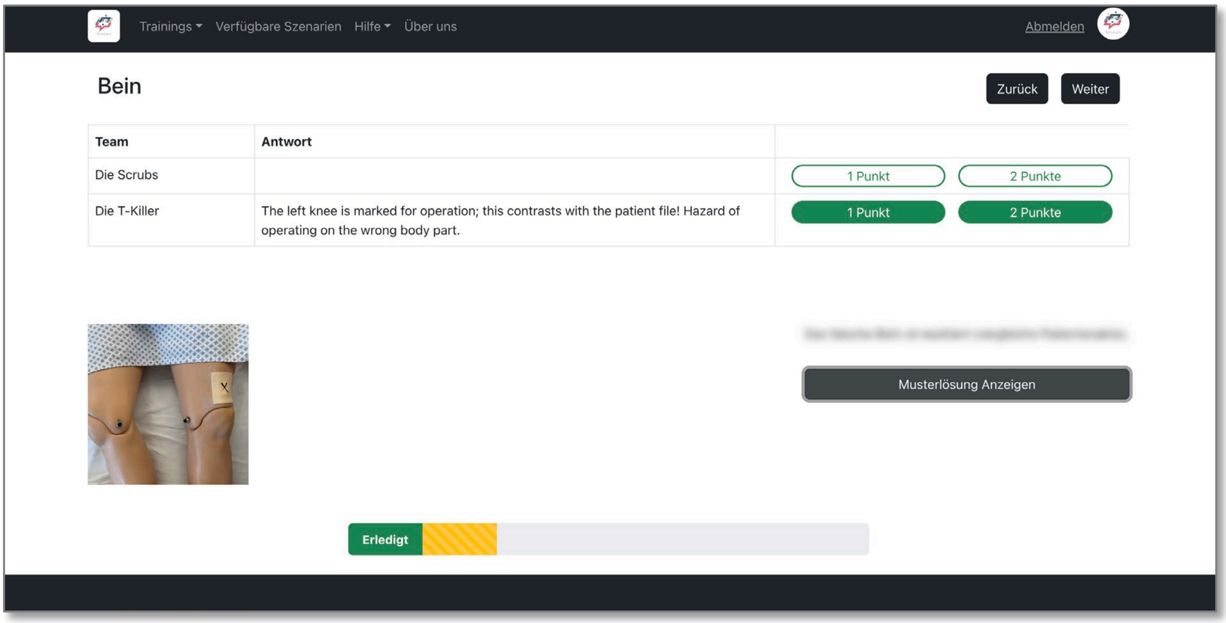

The debriefing follows a structured format, beginning with a clear identification of the item under review (e.g., "Bein"). It proceeds to display the answers provided by each team, which, in some cases, may be none, indicating that the error was not successfully identified. These answers are subject to point-based ratings based on their quality. In above case, team "T-Killer" was awarded 1 point for its result. Additionally, a progress bar tracks the number of errors and patient safety hazards discussed during the debriefing.

Clicking "*Musterlösung anzeigen*" (reveal sample solution) unveils the concealed patient safety hazard, making it available for further discussion.

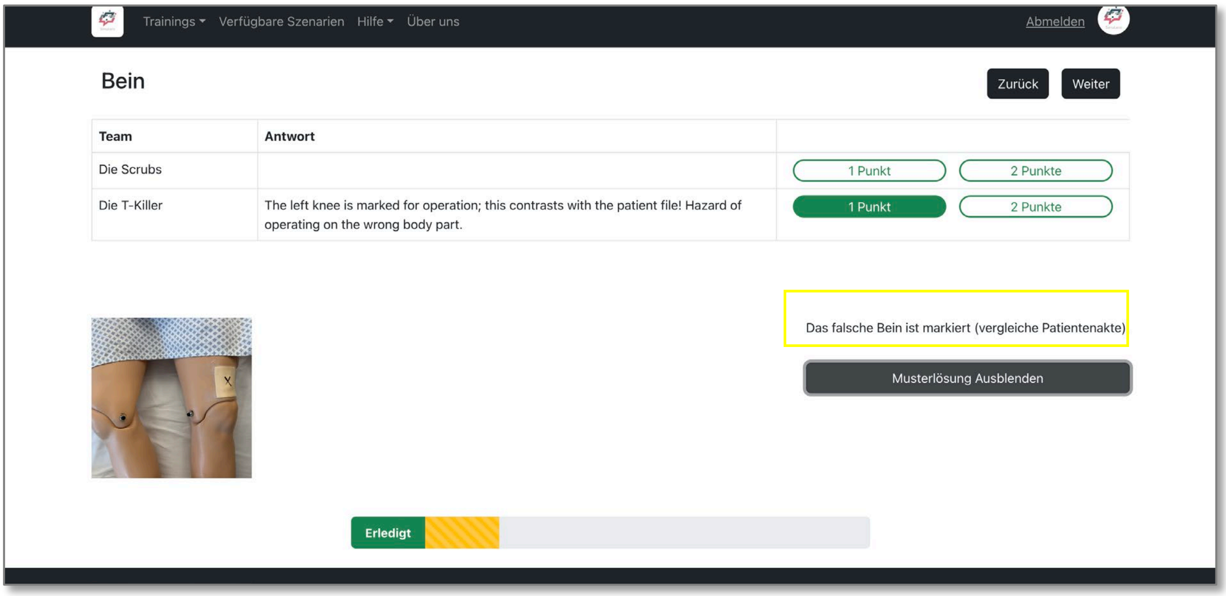

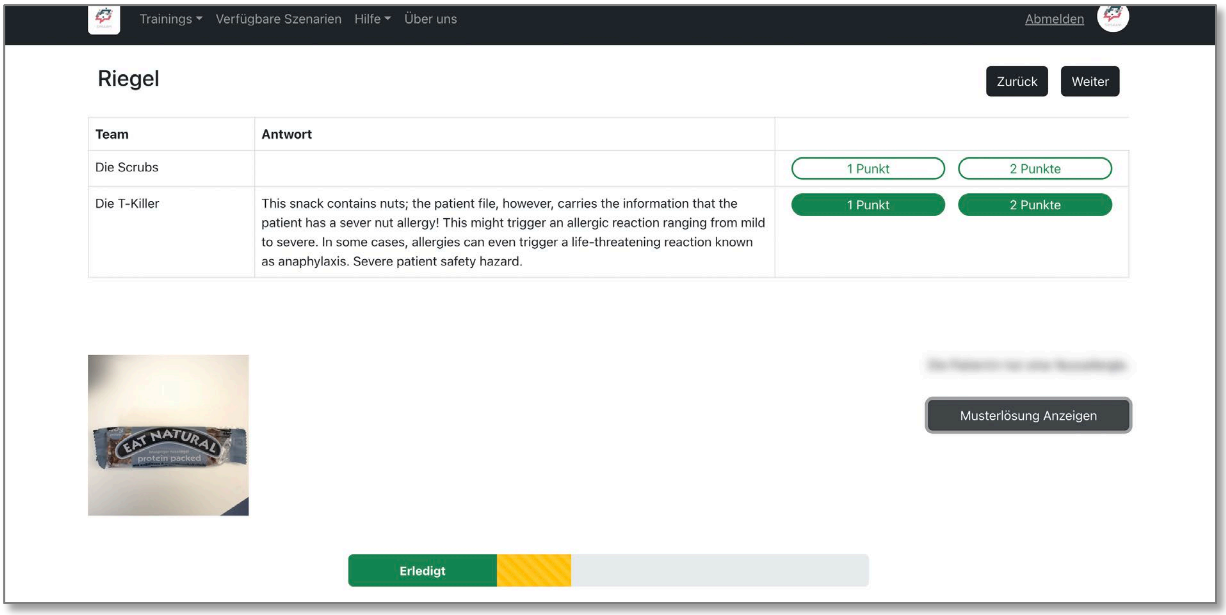

In this case, the team "T-Killer" was awarded 2 points based on their correct and detailed description of both the error and the resulting patient safety hazard.

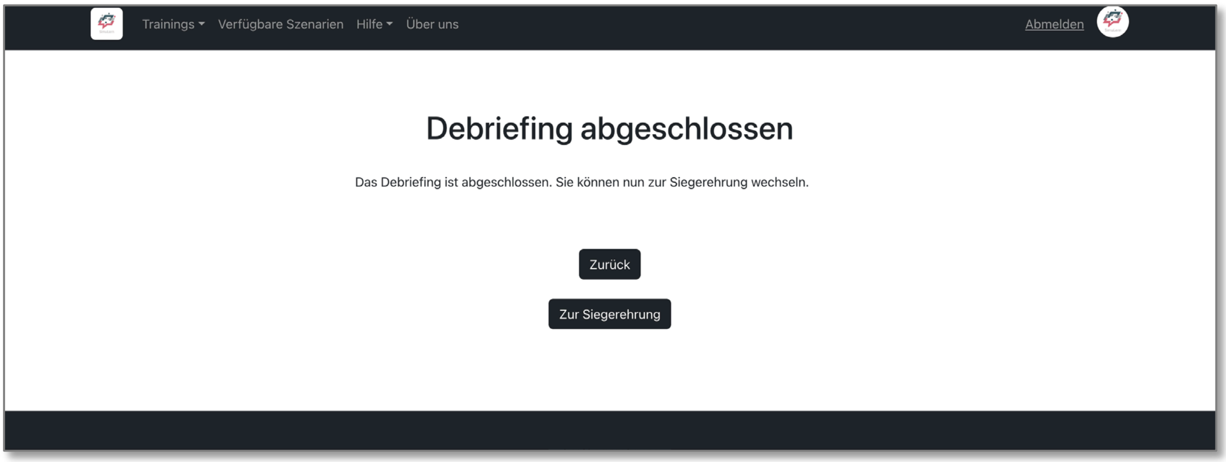

After all included errors in the training scenario have been discussed, the trainer can either go back for further discussion or proceed to award the best teams.

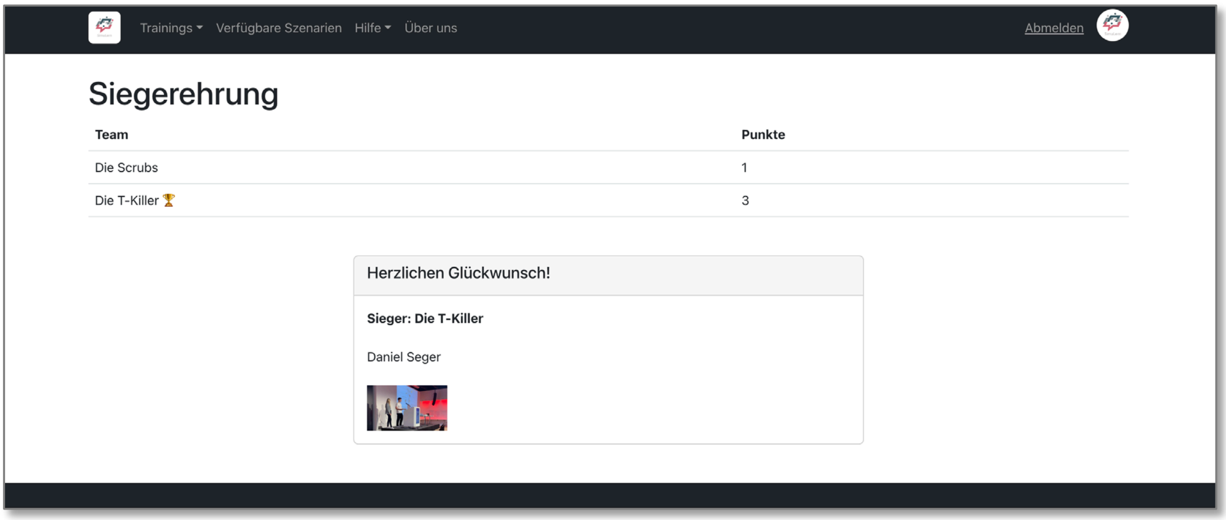

Finally, based on the points awarded during the debriefing session, the three best-performing teams and their respective point totals will be unveiled.

Appendix 11: Application - Virtual scene and 360-degree view

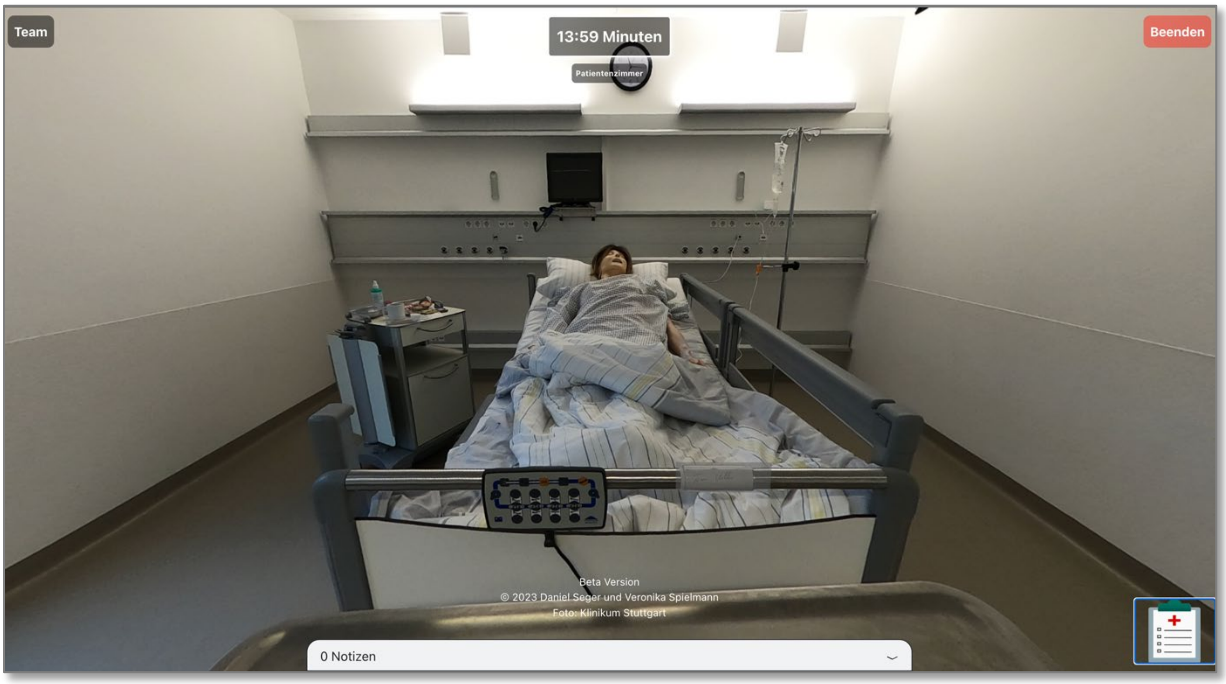

Initial view into the 360-degree "Room of Horrors".

Team members can access their team interface from the top left corner of the screen. In the centre, a countdown timer displays the remaining time to complete the training. Teams can choose to end the training prematurely by clicking the "*Beenden*" (finish) button located in the top right corner. At the bottom centre, there is a shared notebook where team members can document potential errors and patient safety hazards. For additional information, the patient file can be accessed from the bottom right corner.

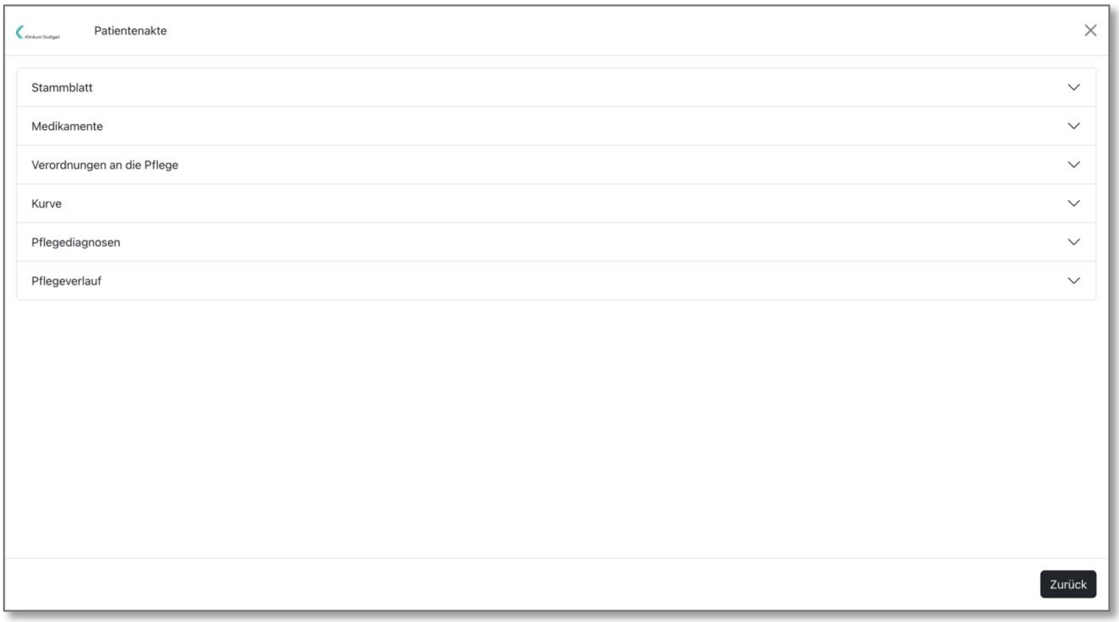

Overview of the patient file.

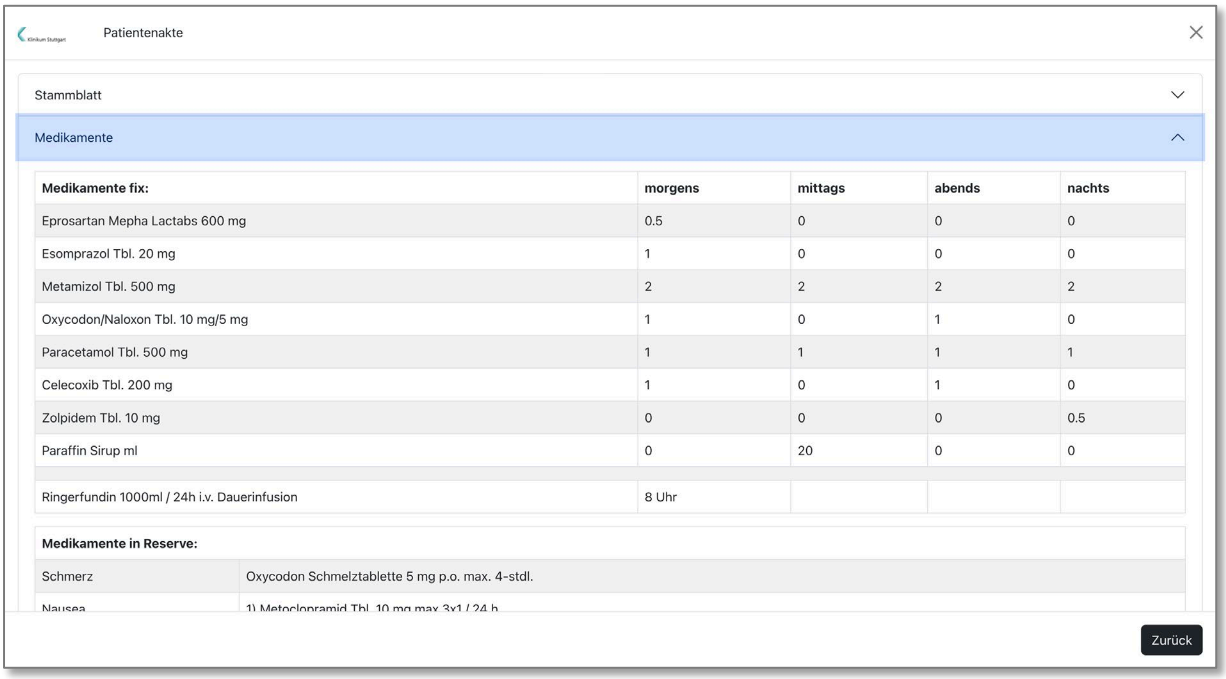

Medikamente fix:	morgens	mittags	abends	nachts
Eprosartan Mepha Lactabs 600 mg	0.5	0	0	0
Esomprazol Tbl. 20 mg	1	0	0	0
Metamizol Tbl. 500 mg	2	2	2	2
Oxycodon/Naloxon Tbl. 10 mg/5 mg	1	0	1	0
Paracetamol Tbl. 500 mg	1	1	1	1
Celecoxib Tbl. 200 mg	1	0	1	0
Zolpidem Tbl. 10 mg	0	0	0	0.5
Paraffin Sirup ml	0	20	0	0
Ringerfundin 1000ml / 24h i.v. Dauerinfusion	8 Uhr			

Medikamente in Reserve:	
Schmerz	Oxycodon Schmelztablette 5 mg p.o. max. 4-stdl.
Nausea	1) Metoclopramid Tbl. 10 mg max 3x1 / 24 h

Detailed look into the medication part of the patient file.

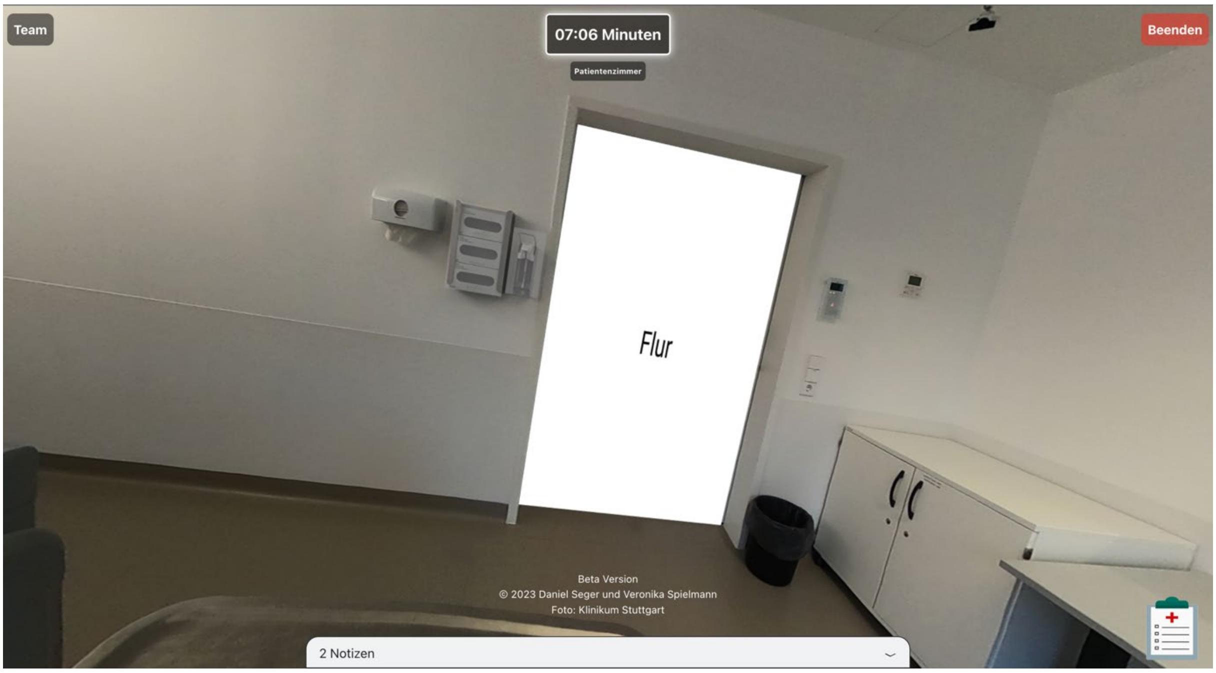

Participants can explore additional rooms by navigating around the virtual environment.

Appendix 12: List of patient safety hazard in the STUPS "Room of Error"

Patient safety hazard		Description of the error	Required preparations
1	Patient accident: risk of falling	Brakes on the hospital bed are not engaged, allowing it to roll	Loosen the bed's brakes
2	Patient accident: risk of falling	Emergency bell is not within the patient's reach	Put the emergency bell out of the patient's reach
3	Clinical processes: Lab tube is prepared for wrong patient	Postoperative blood collection tube carries incorrect patient name (from a different patient)	Label the blood collection tube with a name different from the name in the patient file
4	Medication: false medication	Intravenous infusion contains a 500ml NaCl 0.9% instead of 1000ml Ringer's lactate infusion	Set up an intravenous infusion with 500ml NaCl 0.9%
5	Nutrition: false nutrition	A walnut yogurt is on the patient's side table despite the patient's nut allergy; nut-free food was not prescribed	Put walnut yogurt on the patient's side table
6	Medication: omitted medication	Although the patient file instructs a thrombosis prophylaxis, it was not prescribed	Include the instruction for thrombosis prophylaxis in the patient file
7	Clinical processes: Nursing measures were not carried out despite indication	Patient's pain has not been recorded; patient file does not contain any pain information	Do not include any information on pain in the patient file
8	Documentation: Nursing report for the wrong patient	Case of mistaken patient identity: the nursing report contains the information from a different patient	Write the nursing report for and address it to a different patient
9	Clinical processes: Danger of incorrect operation procedure	The left knee of the patient is marked for operation, although the right knee is to be operated on	Mark the left knee for operation; include the right knee for operation in the patient file
10	Danger of nosocomial infection	Hand disinfectant is empty	Put an empty bottle of hand disinfectant in the holding device
11	Danger of nosocomial infection	Reddening of the skin at the insertion site of the peripheral venous catheter	Display reddening of the skin at the site of injection of the peripheral venous catheter using professional wound make-up
12	Documentation: Missing wrist band	Patient wrist band lies on the table and is not attached to the patient's wrist	Put the patient wrist band loosely on the table
13	Danger of nosocomial infection	No single-use medical gloves are available in the room	Put an empty medical glove box in the holding device

Appendix 13: Application - Virtual scene and clickable error element

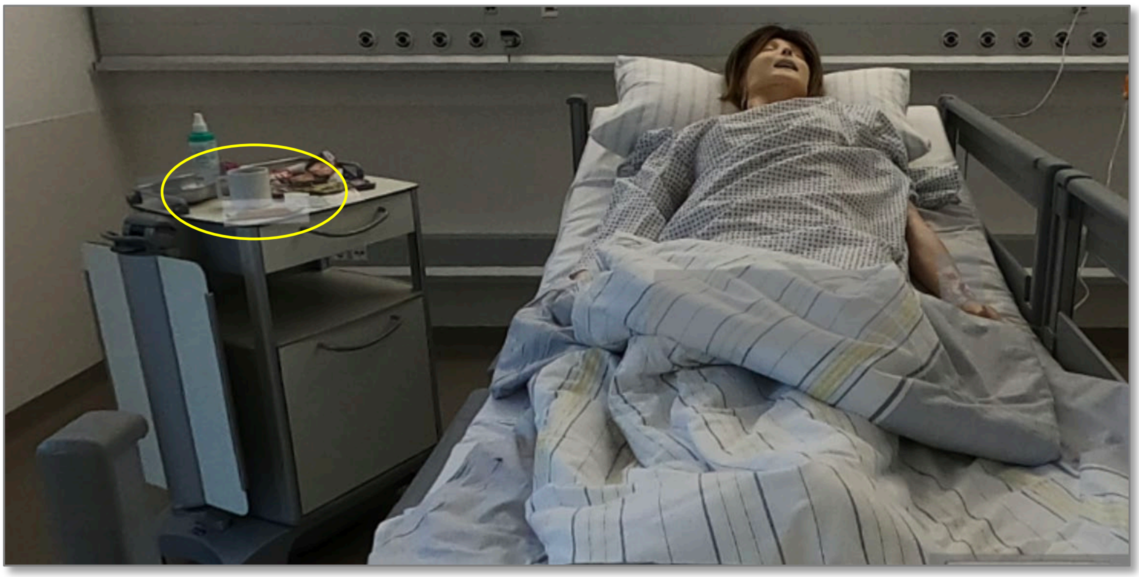

When a user hovers over a clickable element, it becomes highlighted in a light grey shade. Clicking on the element then opens a detailed view.

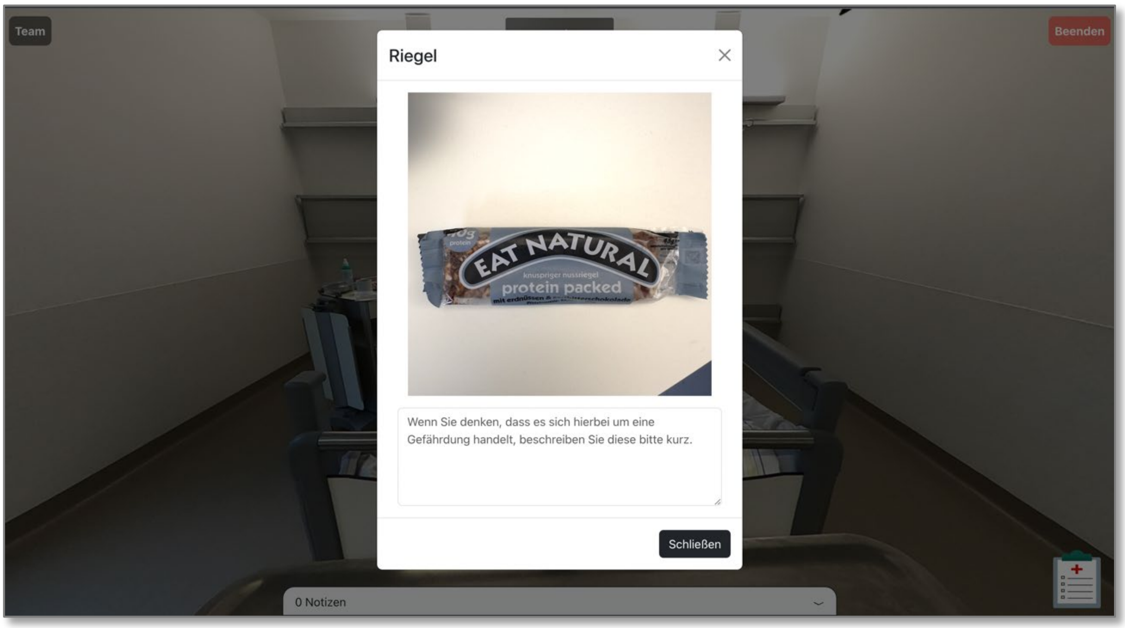

In this screen, team participants can detail potential errors and provide reasoning for the associated patient safety hazards. Their notes are automatically saved in a notebook located at the bottom of the screen and can be modified at any time. During the post-training debriefing session, these saved descriptions in each team's notebook will be revealed, discussed, and compared with the solutions proposed by other teams.

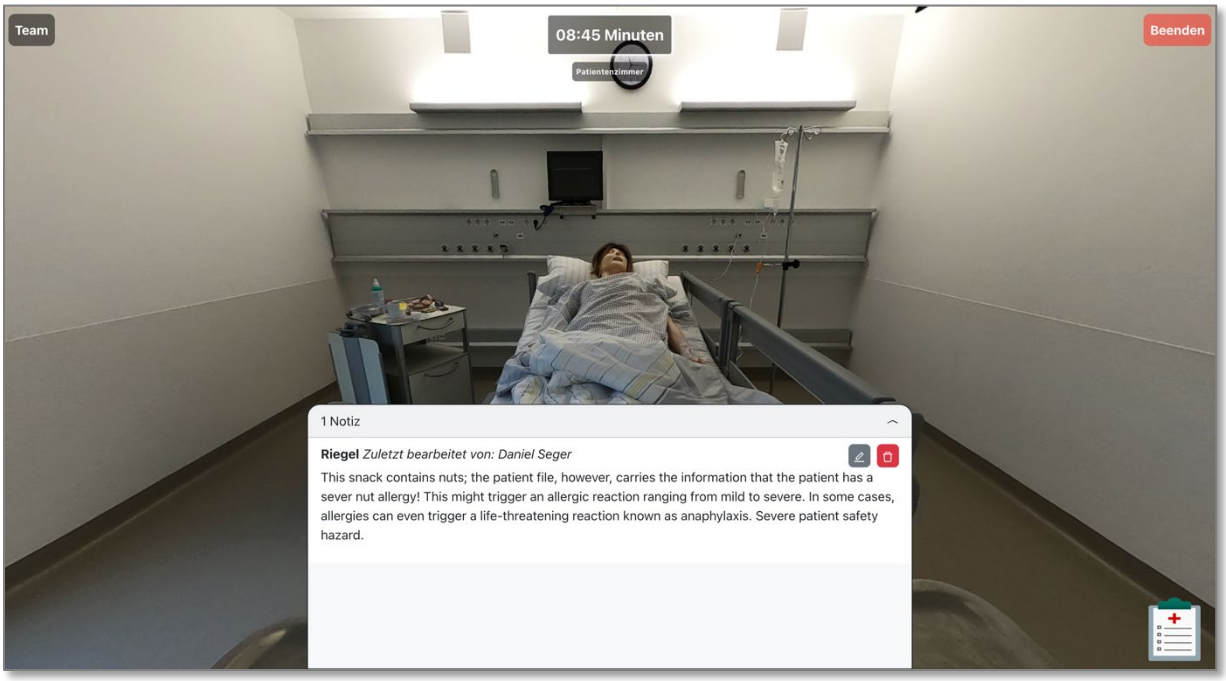

The notebook contains all details regarding errors marked by a team, including the overall number of identified errors, the specification of a certain identified item, the last team member to edit the description, and a detailed account of the associated patient safety hazard. It also provides options to either edit or delete each entry.

Appendix 14: Application - Trainer's view: administer teams

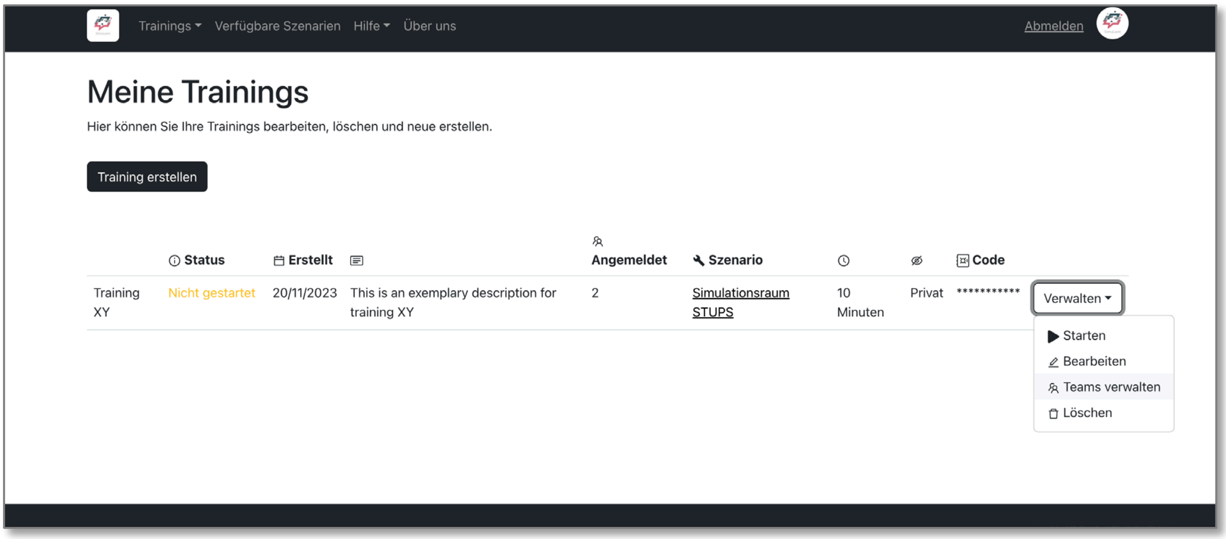

Trainer's dashboard displaying planned trainings, including "Training XY" with 2 registered participants.

Via "*verwalten*" (administer), and "*Teams verwalten*" (administer teams), the trainer can assign the registered participants to interprofessional teams.

Appendix 15: Application - Trainer's view: team assignment and additional teams

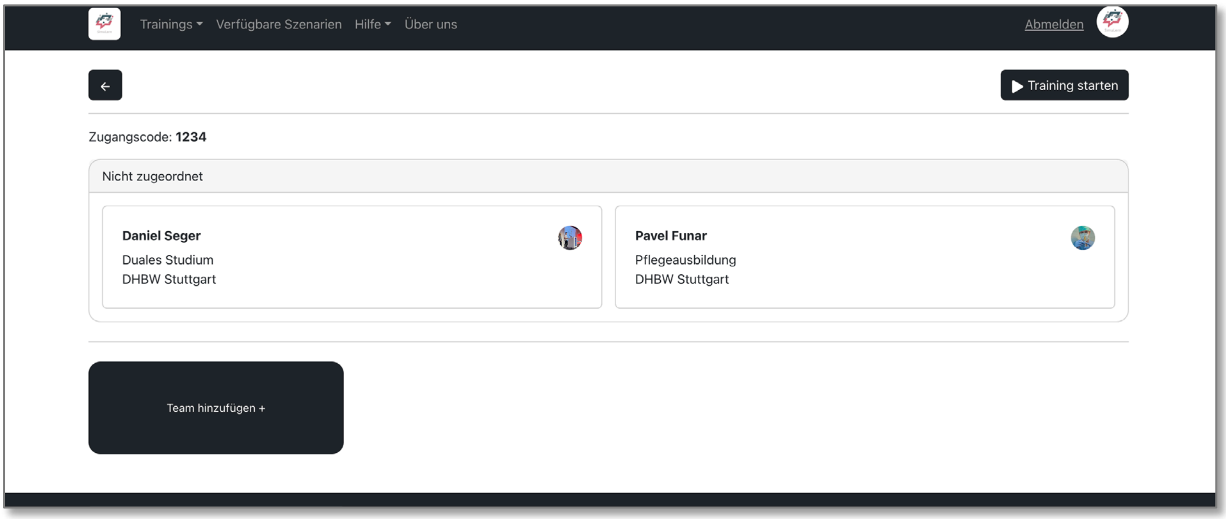

Detailed view for trainers to assign registered participants to teams.
Via "*Team hinzufügen +"* (add team), the trainer can create teams.

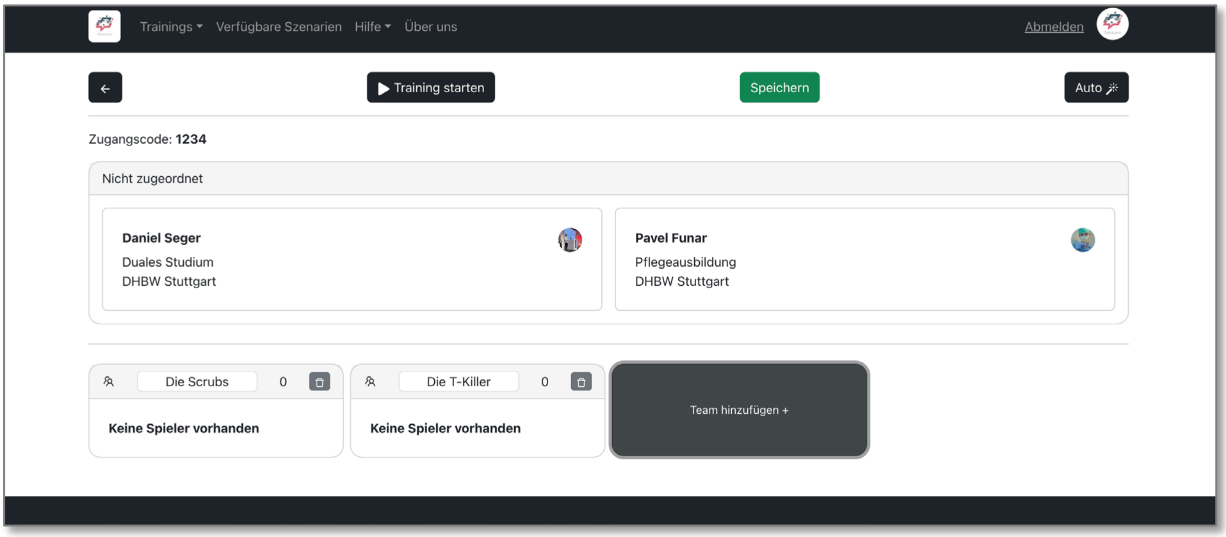

Once the trainer has created the required number of teams, which are assigned default names such as "Die Scrubs" or "Die T-Killer", they can automatically allocate registered participants to these teams by simply clicking the "*Auto*" button. [The number of registered participants in this demonstration here is only two; please note that there are no restrictions to the amount of participants to register or being assigned to teams.]

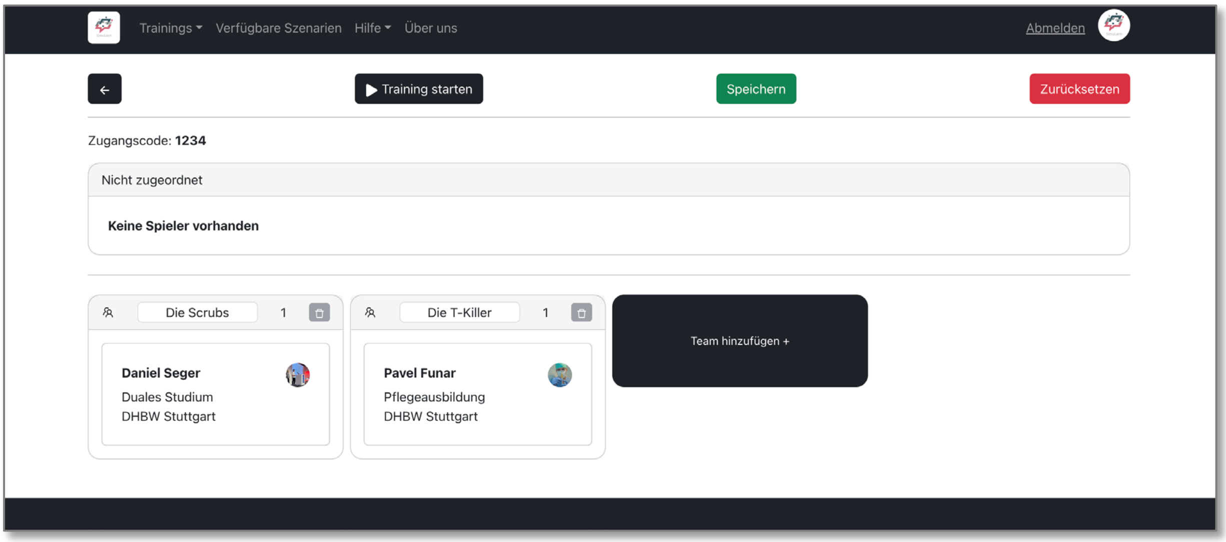

The participants were assigned to different teams. [The demonstration is based on two participants only; to build interprofessional teams, each team should encompass 2-6 members from various professions.] This team assignment and constellation can now be saved or reset by the trainer. As soon as clicking “*Speichern*” (save), participants will be able to view their assigned team in their dashboard.

Attachment 1 to Spielmann V, Ebinger M, Jaki C. *Development of a digital application to train and debrief situational awareness in interprofessional teams: A simulation-based approach to mitigate patient safety hazards with a virtual room of error.* GMS J Med Educ. 2026;43(2):Doc24. DOI: 10.3205/zma001818

Appendix 16: Application - Virtual scene and notification alert

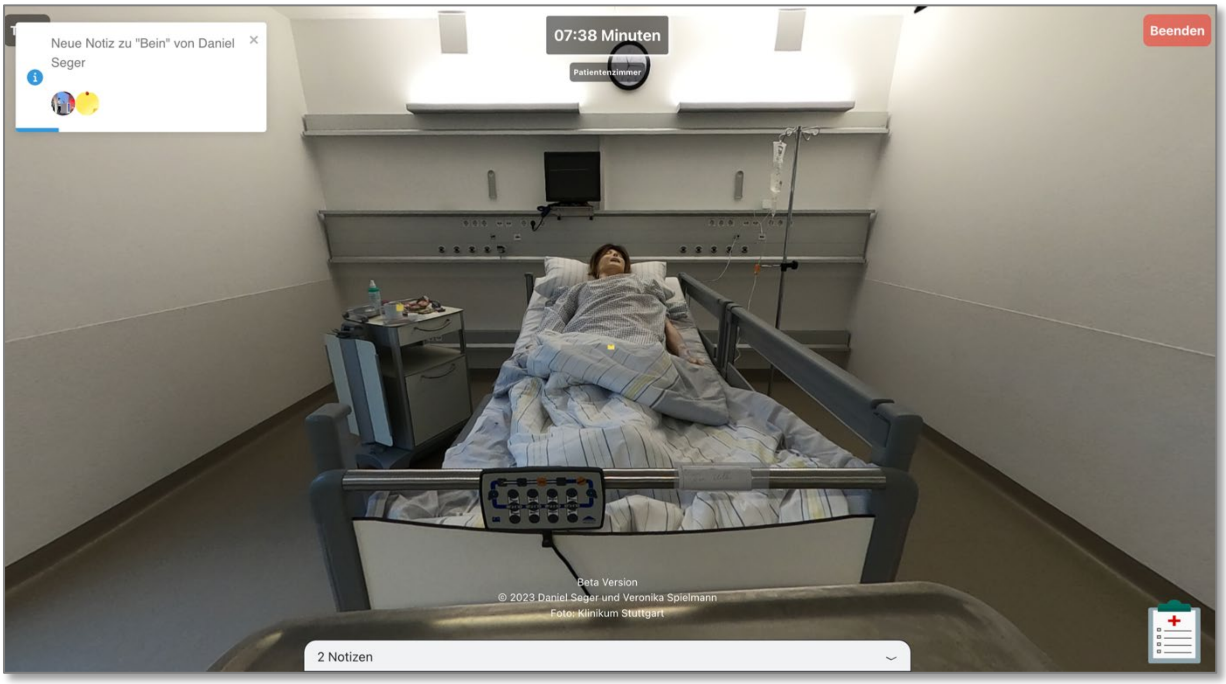

Whenever a team member identifies an error by marking a clickable element and adds a description in the notebook, all other team members are immediately notified through a live update alert, which appears in the top left corner of their screen.

Errors that have already been marked can be easily identified by the small, yellow Post-it notes attached to them.
